# The mitochondrial Ca^2+^ channel MCU is critical for tumor growth by supporting cell cycle progression and proliferation

**DOI:** 10.3389/fcell.2023.1082213

**Published:** 2023-06-08

**Authors:** Emily Fernandez Garcia, Usha Paudel, Michael C. Noji, Caitlyn E. Bowman, Anil K. Rustgi, Jason R. Pitarresi, Kathryn E. Wellen, Zolt Arany, Jillian S. Weissenrieder, J. Kevin Foskett

**Affiliations:** ^1^ Department of Physiology, Perelman School of Medicine, University of Pennsylvania, Philadelphia, PA, United States; ^2^ Department of Cancer Biology and Abramson Family Cancer Research Institute, Perelman School of Medicine, University of Pennsylvania, Philadelphia, PA, United States; ^3^ Department of Medicine, Perelman School of Medicine, Cardiovascular Institute, University of Pennsylvania, Philadelphia, PA, United States; ^4^ Division of Digestive and Liver Diseases, Herbert Irving Comprehensive Cancer Center, Vagelos College of Physicians and Surgeons, Columbia University Irving Medical Center, New York, NY, United States; ^5^ Division of Hematology/Oncology, Departments of Medicine and Molecular, Cell and Cancer Biology, University of Massachusetts Chan Medical School, Worcester, MA, United States; ^6^ Department of Cell and Developmental Biology, Perelman School of Medicine, University of Pennsylvania, Philadelphia, PA, United States

**Keywords:** mitochondria, uniporter, calcium, cancer, metabolism, stable isotope tracing

## Abstract

**Introduction:** The mitochondrial uniporter (MCU) Ca^2+^ ion channel represents the primary means for Ca^2+^ uptake by mitochondria. Mitochondrial matrix Ca^2+^ plays critical roles in mitochondrial bioenergetics by impinging upon respiration, energy production and flux of biochemical intermediates through the TCA cycle. Inhibition of MCU in oncogenic cell lines results in an energetic crisis and reduced cell proliferation unless media is supplemented with nucleosides, pyruvate or α-KG. Nevertheless, the roles of MCU-mediated Ca^2+^ influx in cancer cells remain unclear, in part because of a lack of genetic models.

**Methods:** MCU was genetically deleted in transformed murine fibroblasts for study *in vitro* and *in vivo*. Tumor formation and growth were studied in murine xenograft models. Proliferation, cell invasion, spheroid formation and cell cycle progression were measured *in vitro*. The effects of MCU deletion on survival and cell-death were determined by probing for live/death markers. Mitochondrial bioenergetics were studied by measuring mitochondrial matrix Ca^2+^ concentration, membrane potential, global dehydrogenase activity, respiration, ROS production and inactivating-phosphorylation of pyruvate dehydrogenase. The effects of MCU rescue on metabolism were examined by tracing of glucose and glutamine utilization for fueling of mitochondrial respiration.

**Results:** Transformation of primary fibroblasts *in vitro* was associated with increased MCU expression, enhanced MCU-mediated Ca^2+^ uptake, altered mitochondrial matrix Ca^2+^ concentration responses to agonist stimulation, suppression of inactivating-phosphorylation of pyruvate dehydrogenase and a modest increase of mitochondrial respiration. Genetic MCU deletion inhibited growth of HEK293T cells and transformed fibroblasts in mouse xenograft models, associated with reduced proliferation and delayed cell-cycle progression. MCU deletion inhibited cancer stem cell-like spheroid formation and cell invasion *in vitro*, both predictors of metastatic potential. Surprisingly, mitochondrial matrix [Ca^2+^], membrane potential, global dehydrogenase activity, respiration and ROS production were unaffected. In contrast, MCU deletion elevated glycolysis and glutaminolysis, strongly sensitized cell proliferation to glucose and glutamine limitation, and altered agonist-induced cytoplasmic Ca^2+^ signals.

**Conclusion:** Our results reveal a dependence of tumorigenesis on MCU, mediated by a reliance on MCU for cell metabolism and Ca^2+^ dynamics necessary for cell-cycle progression and cell proliferation.

## 1 Introduction

Calcium (Ca^2+^) transfer from the endoplasmic reticulum (ER) to mitochondria promotes bioenergetics and cell survival by stimulating the activities of dehydrogenases that control the flux of carbons through the TCA cycle, namely pyruvate dehydrogenase (PDH), isocitrate dehydrogenase (IDH) and α -ketoglutarate dehydrogenase ( α -KGDH) (Denton and McCormack, 1980). ER-to-mitochondria Ca^2+^ transfer is mediated by Ca^2+^ release from the ER by inositol 1,4,5-trisphosphate receptors (InsP_3_R) and mitochondrial Ca^2+^ uptake via the mitochondrial Ca^2+^ uniporter (MCU) ion channel complex (Rizzuto et al., 1998). Under resting conditions with cytoplasmic free Ca^2+^ ([Ca^2+^]_cyt_) ∼100 nM, MCU-channel open probability is low due to a regulatory mechanism mediated by intermembrane space-localized dimeric MICU1/2 proteins, so-called channel gatekeeping (Mallilankaraman et al., 2012). Increments in [Ca^2+^]_cyt_ adjacent to the pore of an InsP_3_R channel reach concentrations >50 μM (Foskett, 2010), disabling MCU gatekeeping and promoting mitochondrial Ca^2+^ uptake. Notably, increased expression of InsP_3_R subtypes and MCU in certain cancers have emerged as features associated with aggressiveness and poor survival prognosis (Hall et al., 2014; Shi et al., 2015; Tang et al., 2015; Cardenas et al., 2016; Liberti and Locasale, 2016; Tosatto et al., 2016; Mound et al., 2017; Ren et al., 2017; Guerra et al., 2019; Liu et al., 2020; Wang et al., 2020; Miao et al., 2021).

We previously demonstrated that genetic or pharmacological inhibition of InsP_3_R or MCU in tumorigenic cancer cell lines and normal counterparts decreased cellular ATP content, increased NAD^+^/NADH ratio, reduced cell proliferation and activated mTOR-independent autophagy as a cell survival mechanism (Cardenas et al., 2010; Cardenas et al., 2016), emphasizing the importance of ER-to-mitochondria Ca^2+^ transfer to support basal metabolic requirements. Whereas autophagy was sufficient to enable normal cell survival, it was insufficient in cancer cells, which maintained uncontrolled proliferation leading to cell death by necrosis (Cardenas et al., 2016). Survival defects associated with acute reduction of Ca^2+^ signaling from ER-to-mitochondria could be rescued by media supplementation with nucleosides, pyruvate or α-ketoglutarate, emphasizing compromised mitochondrial bioenergetics as the cause of the observed cancer cell death (Cardenas et al., 2010; Cardenas et al., 2020). A similar reliance on low-level constitutive ER-to-mitochondrial Ca^2+^ transfer was observed even in cancer cells with defective oxidative phosphorylation (OXPHOS) because of the Ca^2+^-dependence of TCA cycle dehydrogenases, particularly α -KGDH (Cardenas et al., 2020). Thus, matrix Ca^2+^ plays critical roles in mitochondrial bioenergetics by impinging upon OXPHOS, ATP synthesis and the flux of biochemical intermediates through the TCA cycle.

“Ca^2+^ addiction” may be a novel feature of cancer with promising therapeutic value. In support, genetic silencing of InsP_3_R enhanced apoptosis in clear cell renal cell carcinoma (Rezuchova et al., 2019) and colon cancer (Shibao et al., 2010). Genetic silencing of MCU in the breast cancer cell line MDA-MB-231 reduced cell motility and invasiveness *in vitro*, as well as tumor growth, lymph node infiltration and lung metastasis *in vivo* (Tosatto et al., 2016). Nevertheless, silencing of MCU in the same MDA-MB-231 line did not reduce cell proliferation, viability or clonogenic survival of cells exposed to different cell stressors (Curry et al., 2013; Hall et al., 2014). Destabilization of the interaction between MCU and MICU1 led to increased cell proliferation and tumor growth of lung cancer (Marchi et al., 2019), yet silencing of MICU1 in ovarian cancer cells enhanced sensitivity to cell-death stimuli and decreased cell migration (Chakraborty et al., 2017). Whereas genetic deletion of InsP_3_R in HEK293 and HeLa cells was found to generate a bioenergetic crisis associated with reduced cell proliferation, genetic deletion of MCU failed to phenocopy this effect (Young et al., 2022). Thus, the roles of ER-to-mitochondria Ca^2+^ transfer in cancer remain unclear.

Lack of genetic models has limited our understanding of the specific roles of MCU in cancer cell biology. Accordingly, we developed *in vitro* transformation models to investigate the role of MCU on the tumorigenic properties of transformed fibroblasts *in vitro* and tumor progression *in vivo*. Our results reveal a fundamental dependence of tumorigenesis *in vivo* and *in vitro* on MCU, mediated by a reliance on mitochondrial Ca^2+^ for cellular metabolism and Ca^2+^ dynamics necessary for cell-cycle progression and cell proliferation.

## 2 Materials and methods

### 2.1 Cell culture

HEK293T, HEK293T MCU-KO (generous gift from Vamsi Mootha, Harvard Medical School, Boston, MA), HEK293T MCU-rescue, immortalized fibroblasts, transformed fibroblasts and MCU-KO transformed fibroblasts were cultured in Dulbecco’s modified Eagle’s medium (DMEM, Mediatech, MT10013CM) supplemented with 10% fetal bovine serum (FBS, HyClone, SH30071.03) and 1% antibiotic-antimycotic (anti-anti, Invitrogen, 15240062), and incubated in a humidified incubator at 37°C with 95% air/5% CO_2_. MCU-KO negative control (NC) transformed fibroblasts and MCU-rescue transformed fibroblasts were cultured in DMEM (10% FBS, 1% anti-anti) and 150 μg/ml Hygromycin B (Mediatech, MT30-240-CR), and incubated in a humidified incubator at 37°C with 95% air/5% CO_2_.

### 2.2 Western blotting

Cells in culture were washed with 1X Dulbecco’s phosphate-buffered saline (DPBS, Mediatech, MT21-031-CM), detached with 0.25% trypsin (Invitrogen, 15090046) and resuspended in DMEM (10% FBS, 1% anti-anti). The cell suspension was washed twice with 1X DPBS and lysed with RIPA buffer [50 mM Tris-HCl (pH 7.5), 150 mM NaCl, 1% NP-40, 0.25% deoxycholic acid, 1 mM EDTA] supplemented with 200 μM phenylmethylsulfonyl fluoride (PMSF) and protease inhibitor cocktail (Roche, 11697498001). For cell lysis, samples were placed in a tube rotator for 30 min at 4°C. Lysates were centrifuged for 10 min. Protein concentration was determined using the Pierce BCA Protein Assay kit (Thermo Scientific, 23227). Samples were prepared for loading with 4X LICOR loading buffer (LICOR, 928-40004) and β-mercaptoethanol. Samples were boiled at 100°C for 5 min. Gel electrophoresis was performed in 4%–12% Bis-Tris gels (NuPAGE, NP0322) and MOPS running buffer (Novex, NP0001). Transfer used nitrocellulose membranes (Sigma, RPN303D) in 20% MeOH Tris/Glycine buffer for 1 h at 100 V. Immunoblotted membranes were blocked with TBS Odyssey blocking buffer (LICOR, 927-50100) for 1 h and then incubated in primary antibodies overnight at 4°C. Membranes were washed 3 times with 0.1% Tween-20 (Bio-Rad, 1706531) TBS (TBS-T) for 5 min and incubated with IRDye secondary antibodies for 1 h in the dark. Blotted membranes were washed 3 times with TBS-T for 5 min and imaged using the Odyssey CLx system. Relative levels of MCU, PDH, pPDH, Tim23, and HSP60 were normalized to tubulin expression detected on the same blots. The antibodies used were: anti-Tubulin (1:5,000, Invitrogen, 322600), anti-MCU (D2z3B) (1:5,000, Cell signaling, 14997s), anti-Pyruvate Dehydrogenase E1-alpha subunit (1:5,000, Abcam, ab110334), anti-phospho-PDHE1-A type I (Ser293) (1:5,000, Millipore, ABS204), anti-Hsp60 (1:5,000, Abcam, ab46798), anti-Tim23 (1:5,000, BD Biosciences, 611223), IRDye 680RD goat anti-mouse (1:10,000, LICOR, 925-68070), IRDye 680RD goat anti-rabbit (1:10,000, LICOR, 926-32211), IRDye 800CW goat anti-mouse (1:10,000, LICOR, 926-68070), and IRDye 800CW goat anti-rabbit (1:10,000, LICOR, 925-3211).

### 2.3 Measurements of mitochondrial Ca^2+^ uptake and membrane potential in permeabilized cell suspensions

Cells in culture were washed with 1X DPBS, detached and resuspended in DMEM (10% FBS, 1% anti-anti). The cell suspension was incubated for 10 min in DMEM (10% FBS, 1% anti-anti) in a humidified incubator at 37°C with 95% air/5% CO_2_, and then centrifuged. The pellet was resuspended in Ca^2+^-free extracellular-like buffer (ECM: 20 mM HEPES-NaOH, 120 mM NaCl, 5 mM KCl, 1 mM KH_2_PO_4_, 0.2 mM MgCl_2_, 0.1 mM EGTA, pH 7.4) made using dH_2_O treated with BT Chelex^®^ 100 resin (Bio-Rad, 143-2832) and incubated for 10 min in a humidified incubator at 37°C with 95% air and 5% CO_2_. The cell suspension was centrifuged and resuspended in Ca^2+^-free intracellular-like buffer (ICM: 20 mM HEPES-NaOH, 10 mM NaCl, 120 mM KCl, 1 mM KH_2_PO_4_, 5 mM succinate, pH 7.5) made using dH_2_O treated with BT Chelex^®^ 100 resin. Fluorescence was monitored in a fluorimeter with multiwavelength excitation and emission (Delta RAM, PTI) at a constant temperature of 37°C. Fura-FF (AAT Bioquest, 21028, Kd = 5.5 μM) excited at 340 nm and 380 nm was monitored at 535 nm emission. TMRE (Molecular Probes, T669) was excited at 560 nm and emission monitored at 595 nm. Addition of reagents during fluorometric measurements was performed according to the following timeline: T = 0, ICM-cell suspension; T = 25 s, 1 μM Fura-FF and 10 nM TMRE; T = 50 s, 0.004% digitonin; T = 100 s to permeabilize the plasma cell membrane, 2 μM thapsigargin (Sigma, T9033) to inhibit cytosolic Ca^2+^ clearance by the ER; T = 200 s, 10 μM CGP37157 (Tocris, 1114) to inhibit Ca^2+^ extrusion from mitochondria; T = 400 s, 3–5 μM CaCl_2_; T = 600 s, 2 μM CCCP; T = 700 s, 1 mM EGTA; and T = 750 s, 1 mM CaCl_2_ (Sigma-Aldrich, 21115). To determine cytosolic (bath) Ca^2+^ concentration ([Ca^2+^]_cyt_) based on the ratiometric calibration of Fura-FF, we used the following equation:

$${\left[{Ca}^{2+}\right]}_{cyt}=\left(\frac{R-R_{\min}}{R_{\max}-R}\right)*\left(\frac{{Sf}_{2}}{{Sb}_{2}}\right){*K}_{d}$$

where R is the ratio of Fura-FF fluorescence at 340/380 nm excitation $;\;R_{\min}$ is the R measured with [Ca^2+^ ] = 0; $R_{\max}$ is the R measured at saturating [Ca^2+^]; ${Sf}_{2}$ is fluorescence of Fura-FF excited at 380 nm at [Ca^2+^ ] = 0 . ${Sb}_{2}$ is fluorescence of Fura-FF excited at 380 nm at saturating [Ca^2+^]; $K_{d}$ is the dissociation constant of Fura-FF $\left(K_{d}\right.$ = 5.5 μM), which was experimentally validated using a set Ca^2+^ calibration buffers (World Precision Instruments, Calbuf-2). Rates of mitochondrial Ca^2+^ uptake were determined by fitting a single exponential from the initial peak after Ca^2+^ addition (T = 400 s) until a steady state was reached (T = 550 s). To determine the mitochondrial membrane potential (Δψ_m_), fluorescence of TMRE was normalized to the maximal value obtained after the addition of CCCP. Then, normalized values between T = 150 s to T = 200 s were averaged to obtain the reported Δψ_m_.

### 2.4 Isolation of primary mouse fibroblasts

Primary mouse fibroblasts were isolated from the skin of 1-week old homozygous Mcu^fl/fl^ pups (The Jackson Laboratory, stock #029817). Pups were euthanized by decapitation. The skin was washed three times with ice-cold 1X DPBS (1% anti-anti) and sliced into 1-cm pieces using a clean razor blade. Tissue was placed in pre-warmed 500 μg/ml thermolysin solution (Sigma, T7902) prepared in HEPES buffer (Sigma, H3375) and incubated for 2 h at 37°C with constant agitation. The tissue was transferred to a pre-warmed 0.125 U/mL collagenase solution (Collagenase Type 2, Worthington Biochemical, LS004176) prepared in DMEM (10% FBS, 1% anti-anti) and incubated for 2 h at 37°C with constant agitation. Digested tissue was forcefully ground using a 10 ml syringe plunger and centrifuged. The fibroblast-containing pellet was plated in DMEM (10% FBS, 1% anti-anti). To validate the identity of the isolated primary fibroblast population, the cells were sorted by fluorescence-activated cell sorting (FACS) for the surface marker platelet-derived growth factor receptor alpha (PDGFRA), using antibodies anti-CD140a (PDGFRA) Monoclonal Antibody (APA5) (1:100, Thermo Fisher, 14-1401-81) and goat anti-Rat IgG H&L (Alexa Fluor^®^ 488) (1:200, Abcam, 150157), and emission filter 530 nm. HEK293T and human fibroblasts were used as negative and positive controls, respectively.

### 2.5 Generation of lentivirus for immortalization and transformation of primary fibroblasts

Plasmids for the expression of pRRL-TERT, constitutively-active pRRL-CDK4^R24C^, dominant-negative pRRL-TP53^R248W^, and pRRL-HRas^G12V^ were generated and kindly donated by Dr. Todd W. Ridky. Lentivirus was produced in HEK293T cells. A day before transfection, HEK293T cells were seeded in 15-cm dishes, aiming for 50% confluency the next day. 24 h after seeding, cells were co-transfected with 22.5 μg of lentiviral plasmid, 16.9 μg of the packaging plasmid psPAX2 (Addgene, 12260) and 5.6 μg of the envelope plasmid VSV-G (Addgene, 14888) using Lipofectamine 3000 (Thermo Fisher, L3000015). After 16 h, 10 mM Na-butyrate was added to the culture. Lentivirus-containing media was collected after 48 and 72 h of transfection and filtered through a 45 μm filter.

### 2.6 Generation of immortalized and transformed primary fibroblast cell lines

Primary fibroblasts isolated from 1-week old Mcu^fl/fl^ mouse pups were immortalized by sequential transduction with TERT and CDK4^R24C^ lentiviruses. Immortalized cells were kept in culture as immortalized cells or transduced with p53^R248W^ and HRas^G12V^ lentiviruses for oncogenic transformation. Genomic elimination of MCU (MCU-KO) in transformed fibroblasts was achieved by transient expression of Cre recombinase by transfection with the plasmid pLM-CMV-R-Cre (Addgene, 27546), which codes for mCherry-Cre recombinase, using Lipofectamine 3000. The cells were sorted by FACS for red mCherry fluorescence. Collected cells were diluted and seeded at one cell/well in 96-well plates. Colonies were grown and verified by Western blot for MCU-KO. Rescue of MCU (MCU-rescue) was achieved by transfection of MCU-KO transformed fibroblasts with the plasmid pCMV3-MCU-FLAG (Sino Biological, MG5A1846-CF), which codes for MCU, using Lipofectamine 3000. Generation of MCU-KO NC transformed fibroblasts was achieved by transfection of MCU-KO transformed fibroblasts with the plasmid pCMV3-C-FLAG (Sino Biological, CV012), using Lipofectamine 3000. Transfection of MCU-KO NC and MCU-rescue was followed by Hygromycin B selection and isolation of single clones in 96-well plates. Colonies were grown and verified by Western blot for MCU expression.

### 2.7 Mouse tumor xenografts

All animal procedures were approved by the Institutional Animal Care and Use Committee of the University of Pennsylvania (protocol #806559). Tumor xenografts of primary mouse fibroblasts were performed in 4–8 week old male outbred athymic nude mice J:Nu (Jackson Laboratory, stock #007850). Tumor xenografts of HEK293T were performed in 4–8 week old male immunodeficient mice (NOD.*Cg-Prkdc^scid^*/J) (Jackson Laboratory, stock # 001303). Mice were housed in pathogen-free conditions in a 12 h light/12 h dark cycle with food and water *ad libitum*. The day of surgery, fibroblasts were washed with 1X DPBS, trypsinized and resuspended in DMEM (10% FBS, 1% anti-anti). For immortalized vs. transformed xenograft experiments, cells were resuspended at a density of 10 × 10^6^ cells/mL. For HEK293T xenograft experiments, cells were resuspended at a density of 10 × 10^6^ cells/mL. For transformed vs. transformed MCU-KO xenograft experiments, cells were resuspended at a density of 40 × 10^6^ cells/mL. Mice were anesthetized using 1.5%–4% isoflurane in an induction chamber. Just before injection, cells were mixed with Matrigel (Fisher Scientific, 354234) in equal parts. Injection was done into the subcutaneous space of the mouse flanks. Each mouse received one injection into the subcutaneous space of one flank and another of immortalized or MCU-KO fibroblasts into the opposite flank. For HEK293T xenografts, each mouse received one injection of HEK293T-WT cells into one flank and another of HEK293T MCU-KO or HEK293T MCU-rescue into the opposite flank. After injections, tumor formation and progression were monitored for 3–4 weeks and measured with a caliper. Tumor volume was determined using the following equation:

$$Tumor\;volume\;\left({mm}^{3}\right)=\left(\frac{D_{long}}{{D_{short}}^{2}}\right)÷2$$

where $D_{long}$ is the longest diameter and $D_{short}$ is the shortest diameter of the tumor. At the end of the experiment, the mice were euthanized by CO_2_ exposure followed by decapitation. Tumors were excised and formalin fixed for further processing. In brief, tumors were submerged in zinc formalin solution for 24 h at 4°C and then transferred into 70% ethanol. Tumors were embedded in paraffin (Fisher Scientific, T56-5), cut into 5-μm sections, and mounted by the Molecular Pathology and Imaging Core of the Perelman School of Medicine at the University of Pennsylvania.

### 2.8 Immunostaining and quantification

Immunostaining was performed by the Skin Histology and Characterization Core of the Perelman School of Medicine at the University of Pennsylvania. Tumor sections were imaged at ×20 using a Keyence B2-X710 microscope at eight different regions of heterogeneous tissue excluding necrotic areas, the centers and tumor edges. Each region was imaged at 20 continuous field of views that were stitched to create a single image. Regions were imaged with three different channels: blue (DAPI), red (Ki-67), and green (TUNEL) and saved as a composite. Images were imported into the software GNU Image Manipulation Program (GIMP, www.gimp.org) and the fluorescent signal of each channel was extracted as a greyscale component. Then, images were imported into the software FIJI (Schindelin et al., 2012). A signal index was determined by summing the pixel counts of the fluorescence signals as previously described (Billings et al., 2015; Asrani et al., 2019). In brief, greyscale components were transformed into binary images where the number of black pixels, representative of fluorescence, were quantified to obtain a DAPI, Ki-67, and TUNEL index. Using the histogram analysis tool in FIJI, each pixel was cataloged as background or signal and then summed to obtain total number of pixels, index of background, and index of signal. As a complementary approach to quantify individual Ki-67^+^ cells in tumor sections, each field of view of a region imaged with the red channel was individually processed in FIJI. First, the background was subtracted, then the image was converted into a binary component. Finally, each nucleus was represented as a single particle and counted to obtain the total number of Ki-67^+^ cells.

### 2.9 Histochemistry and histology

Tumor-section slides were processed for hematoxylin and eosin (H&E) staining by the Molecular Pathology and Imaging Core of the Perelman School of Medicine at the University of Pennsylvania. In brief, tumor sections were deparaffinized in xylene (Azer, ES609), rehydrated in ethanol (Azer, ES631) and washed with deionized water. Then, tissue was stained with hematoxylin (Leica Biosystems, 3801540) followed by eosin (Leica Biosystems, 3801600). Tissue was dehydrated with alcohol and mounted. Each slide was manually scored under the supervision of a pathologist (Dr. John T. Seykora). Necrosis analysis was done by measuring normal tissue area and necrotic area in the whole section using an eyepiece graticule with calibrated grids (25 mm). For scoring mitotic figures and giant multinucleated cells, each slide was examined at ×40 at 10 different regions of heterogeneous tissue excluding necrotic areas, the centers and tumor edges. Representative images of necrotic and normal tumor tissue, mitotic figures, and giant multinucleated cells were taken at ×20 using a Keyence B2-X710 microscope.

### 2.10 Sphere formation assay

Cells in culture were washed with 1X DPBS, trypsinized and resuspended in tumorsphere media, composed of DMEM/F12 medium (Sigma-Aldrich, D8437) supplemented with 0.4% bovine serum albumin (BSA, Life Technologies, Invitrogen, 15561020), 1% anti-anti, 20 ng/ml epidermal growth factor (Sigma-Aldrich, E5036), 10 ng/ml basic fibroblast growth factor (Sigma-Aldrich, F0291), 5 µg/ml insulin (Life Technologies, Invitrogen, A11429IJ) and 1X B27 supplement (Life Technologies, Invitrogen, 17504-044), as previously described (Johnson et al., 2013; Weissenrieder et al., 2020). Briefly, cells were plated at a density of 200 cells/well in 200 µL media in low adhesion plates, generated by applying Aggrewell solution (Stemcell Technologies, 7010) for 5 min, spinning down for 5 min at 1,300 rpm and then rinsing gently with basal media (DMEM/F12, 0.4% BSA, 1% anti-anti). To reduce evaporation, outside wells were filled with sterile DPBS and not used as experimental wells. Quantification of spheroids, defined as rounded aggregates of cells with a smooth surface and poor cell-to-cell definition, was performed after 7 days of incubation.

### 2.11 Proliferation assay

Cells in culture were washed with 1X DPBS, trypsinized and resuspended in DMEM (10% FBS, 1% anti-anti). Cells were plated at a density of 15,000 cells/well at day 0. Quantification of the number of cells per well was performed for 4 days-post-seeding without continuous passage. For the proliferation assay in low nutrient conditions, cells were plated at a density of 15,000 cells/well at day 0 in high glucose (25 mM) and glutamine (4.5 mM) media. Then, at day post-seeding one media was changed to low glucose (4.5 mM) and glutamine (0.75 mM) media. Accordingly, quantification of the number of cells per well was performed for 4 days post-seeding without continuous passage.

### 2.12 Transwell invasion assay

Cells in culture were washed with 1X DPBS, trypsinized and resuspended in DMEM (10% FBS, 1% anti-anti). Cells were plated at a density of 15,000 cells/well atop of 10 mg/ml Matrigel coated Transwells (Corning, 3464) in serum-free DMEM, while the bottom well contained 10% FBS DMEM. After 24 h incubation, cells at the bottom of the membrane were fixed with 4% PFA (Electron Microscopy Sciences, 15713) and stained with 1 μg/μL Hoechst 33342 (Invitrogen, H3570). Quantification was done by analyzing three different fields of views of each well, imaged at ×20, and quantified automatically by the software FIJI.

### 2.13 Annexin V/DAPI FACS and cell viability analysis

Cells in culture were washed with 1X DPBS, trypsinized and resuspended in DMEM (10% FBS, 1% anti-anti). Cell suspension was centrifuged at 1,000 rpm for 5 min. The pellet was washed with 1X DPBS and centrifuged at 1,000 rpm for 5 min. The supernatant was aspirated and the cell pellet was resuspended and incubated in binding buffer with fluorochrome [488]-conjugated Annexin V (Thermo Fisher Scientific, R37174), according to manufacturer’s instructions. After 15-min incubation, 4 mM DAPI (Abcam, ab228549) was added to the suspension and cells were analyzed by FACS with emission filters 530 and 440 nm for Annexin V/DAPI detection. Data were plotted and analyzed using FCS Express software. Debris was excluded by a first gate set in forward-area and side-area (FSC-A vs. SSC-A) plots. Doublets were excluded by a second level of gating in forward-area and forward-height (FSC-A vs. FSC-H) plots. Cells within gates were plotted in DAPI vs. Annexin V plots. Based on negative and 1 μM staurosporine-treated controls for each FACS plot, gated cells were divided into four quadrants: lower left (healthy cells), lower right (early apoptotic cells), upper right (late apoptotic/dead cells), and upper left (necrotic cells). Final values represent percent of gated cells in each quadrant.

### 2.14 Measurement of oxygen consumption rates (OCR)

Cells in culture were washed with 1X DPBS, trypsinized and resuspended in DMEM (10% FBS, 1% anti-anti), plated at a density of 30,000 cells/well and incubated for ∼16 h. Media was changed to DMEM base assay medium (Sigma, D5030) supplemented with 1 mM Na-pyruvate, 2 mM glutamine, and 10 mM glucose. Baseline OCR was measured three times before the addition of 2 μM oligomycin, followed by 0.5 μM FCCP, and finally 0.5 μM rotenone/antimycin A+ 1 μg/μL Hoechst 33342 (Invitrogen, H3570) (Seahorse XF Cell Mito Stress Test Kit, Agilent, 103015) according to the manufacturer’s instructions. To measure changes in OCR following acute agonist stimulation, baseline OCR determination was followed by the addition of 500 μM ATP. At the end of each assay, the center portion of each well was imaged at ×20 to detect nuclei counterstained with Hoechst. Nuclei were automatically counted using FIJI and used to normalize individual OCR/well.

### 2.15 Measurement of extracellular acidification rates (ECAR)

Cells in culture were washed with 1X DPBS, trypsinized and resuspended in DMEM (10% FBS, 1% anti-anti), plated at a density of 35,000 cells/well and incubated for ∼16 h. Media was changed to DMEM base assay medium (Sigma, D5030) supplemented with 1 mM glutamine. Baseline ECAR was measured three times before the addition of 10 μM glucose, followed by 1 μM oligomycin, and finally 50 μM 2-deoxy-glucose (2-DG) + 1 μg/μL Hoechst (Seahorse XF Glycolysis Stress Test Kit, Agilent, 103020) according to the manufacturer’s instructions. At the end of each assay, the center portion of each well was imaged at ×20 to detect nuclei counterstained with Hoechst. Nuclei were automatically counted using FIJI and used to normalize individual ECAR/well.

### 2.16 XTT assay

Cells in culture were washed with 1X DPBS, trypsinized, resuspended in DMEM (10% FBS, 1% anti-anti) and plated at a density of 30,000 cells/well. After ∼16-h incubation, cells were incubated with XTT (Millipore Sigma, 11465015001) according to the manufacturer’s instructions. Reduction of XTT was quantified by formazan absorption at 490 nm and normalized to reference wavelength absorbance at 690 nm. XTT absorbance was detected every 30 min for 4 h, during which readout is linear, allowing for the best detection of differences between cell lines.

### 2.17 Measurement of reactive oxygen species (ROS)

Cells in culture were washed with 1X DPBS, trypsinized, resuspended in DMEM (10% FBS, 1% anti-anti), and plated at a density of 30,000 cells/well. After ∼16 h incubation, cells were incubated with 25 μM 2′,7′-dichlorodihydrofluorescein diacetate (DCFDA) (Sigma-Aldrich, D6883) and 1 μg/μL Hoechst. DCFDA oxidation was detected at 535 nm and normalized to Hoechst.

### 2.18 Measurement of [Ca^2+^]_mit_

Cells in culture were washed with 1X DPBS, trypsinized, resuspended in DMEM (10% FBS, 1% anti-anti), and seeded onto coverslips. After ∼16 h, the cells were transfected with Lipofectamine 3000 and 2.5 μg of the plasmid pCMV CEPIA2mt (Addgene, 58218) or CEPIA3mt (gift from M. Trebak, Addgene, 58219). Cells were imaged 48 h after transfection. In brief, coverslips were transferred to a perfusion chamber and perfused with extracellular-like solution (135 mM NaCl, 5.9 mM KCl, 1.2 mM MgCl_2_, 11.6 mM HEPES, 10 mM glucose, 1.5 mM CaCl_2_). Fluorescence of CEPIA2/3 mt was monitored using a Nikon Eclipse microscope at 20x at the excitation/emission wavelengths 488/500–550 nm (Suzuki et al., 2014). For each measurement, three images of the same field of view were taken 60 s apart. To calibrate CEPIA2mt signals, cells were perfused with 1 μM ionomycin (Invitrogen, 124222) followed by a 0-Ca^2+^ Tyrode’s solution (135 mM NaCl, 5.9 mM KCl, 1.2 MgCl_2_, 1 mM EGTA, 11.6 mM HEPES) to obtain maximum and minimum intensity of CEPIA2mt. Data analysis was performed using VisiView software (Visitron Systems GmbH). For each field of view, a region of interest (ROI) was delineated around the edges of each cell to obtain basal, maximum, and minimum fluorescence of CEPIA2mt. To determine the basal mitochondrial Ca^2+^ concentration ([Ca^2+^]_mit_), we used the following equation:

$${\left[{Ca}^{2+}\right]}_{mit}=\left(\frac{F-F_{\min}}{F_{\max}-F}\right){*K}_{d}$$

where F is CEPIA2mt fluorescence, $F_{\min}$ is F at 0-Ca^2+^, $F_{\max}$ is F at saturating Ca^2+^, and $K_{d}$ is the dissociation constant of CEPIA2mt ( $K_{d}$ = 0.16 μM) (Suzuki et al., 2014).

### 2.19 Measurement of relative cytoplasmic [Ca^2+^]

Cells were washed with 1X DPBS, trypsinized, resuspended in DMEM (10% FBS, 1% anti-anti), and seeded onto coverslips. After 2 days, cells were incubated with 2 μM Fura-2 AM (Thermo Fisher Scientific, F1225) for 30 min at room temperature. Imaging was performed with a Nikon Eclipse Ti-U microscope using a ×20/0.75 objective. Fura-2 AM ( $K_{d}$ = 140 nM) was excited at 340 nm and 380 nm and monitored at 535 nm emission. Before imaging, cells were perfused with 1.5 mM Ca^2+^ Tyrode’s solution (135 mM NaCl, 5.9 mM KCl, 1.2 MgCl_2_, 1.5 mM CaCl_2_, 11.6 mM HEPES) for 5 min. Baseline recording was done for 1 min while perfusing with 1.5 mM Ca^2+^ Tyrode’s solution. Then, 2 μM ATP was added into perfusion chamber and agonist-induced responses were recorded for 300 s. Recording was terminated after washing the cells with 0-Ca^2+^ Tyrode’s solution (135 mM NaCl, 5.9 mM KCl, 1.2 MgCl_2_, 1 mM EGTA, 11.6 mM HEPES) for 100 s. Raw traces for each cell were obtained using VisiView. For analysis, background fluorescence recorded from a cell-free space in the field of view of each coverslip was subtracted from each image, and the background-subtracted signal was normalized to the averaged baseline value (0—50 s) to obtain the ratio over time (R/R_0_). Responses were classified as oscillating, single peak, and sustained using a series of statistical parameters applied from the end of the initial peak to the end of the ATP stimulation. Those traces in which there was no agonist-induced response, the Fura-2 AM signal was significantly elevated or too low (20%), and where the responses were qualitatively identified as complex or mixed (19%), were not included in the analysis.

### 2.20 Cell cycle analysis

Activated lovastatin was prepared from its inactive lactone prodrug form (Millipore Sigma, PHR1285) as described (Keyomarsi et al., 1991). Cells were washed with 1X DPBS, trypsinized, resuspended in DMEM (10% FBS, 1% anti-anti), and seeded onto 6-well plates. Two days post-seeding, media of all samples, except asynchronous controls, was replaced with 15 μM lovastatin media. After 24 h incubation, lovastatin media was replaced with DMEM (10% FBS, 1% anti-anti). At this moment, asynchronous control and lovastatin-unreleased controls were collected and fixed. The rest of the samples were collected after 24 h of cell cycle release from lovastatin synchronization. For sample collection and fixation, media was collected, the cells were washed with 1X DPBS, trypsinized, and resuspended with the collected media. The cell suspension was centrifuged at 1,000 rpm for 5 min. The supernatant was aspirated and the cell pellet was resuspended in ice-cold 1XDPBS. The cells were then centrifuged at 1,000 rpm for 5 min. The supernatant was aspirated and the cells were resuspended in 100 μL of 1X DPBS. Finally, fixation buffer (80% EtOH in dH_2_O) was added in a drop-wise manner while cells were vortexed. For FACS analysis, cells were stained with 4 mM DAPI (Abcam, ab228549) and analyzed at 440 nm emission. Data were plotted and analyzed using FCS Express. Debris was excluded by a first gate set in FSC-A vs. SSC-A plots. Doublets were excluded by a second level of gating in FSC-A vs. FSC-H plots and verified in a DAPI-W vs. DAPI-A plot. Gated cells were plotted in DAPI vs. cell count histogram. Final values represent the percent of gated cells in each phase (G1, S, G2) of the cell cycle, which were determined using the Multicycle AV DNA analysis software in FCS. Unsynchronized- and lovastatin-synchronized samples were used as controls to validate the G1, S, and G2 peaks identified using Multicycle software for cell cycle analysis.

### 2.21 Measurement of glucose and glutamine uptake, and lactate and glutamate production

Cells in culture were washed with 1X DPBS, detached with 0.25% trypsin, and resuspended in DMEM (10% FBS, 1% anti-anti). Cells were plated at a density of 50,000 cells/well in 6-well plates in a total of 2 mL of culture media. Wells with no cells were kept as controls for normalization of metabolite concentrations in media. After 48 h post-seeding, media was removed, wells were washed with 1X DPBS, and fresh media was added to each well. Samples of culture media were collected 24 h post-media change and stored at −80°C until analysis. For normalization purposes, number of cells per well was determined manually using a hemacytometer. Quantification of glucose, lactate, glutamine, and glutamate concentrations (nM) in samples of cell culture supernatant was determined enzymatically with a bioanalyzer (YSI2950, YSI Incorporated, Yellow Springs, OH, United States). Rate of metabolite consumption ( $v_{c}$ ), rate of metabolite production ( $v_{p}$ ), cell number area under the curve ( A ), and doubling time ( d ) where calculated with the following equations:

$$v_{c}=\frac{{V(x}_{medium\;control}-x_{final})}{A}$$

$$v_{p}=\frac{{V(x}_{final}-x_{medium\;control})}{A}$$

$$A=\frac{N\left(T\right)*d}{\ln2\;\left(1-2-\frac{T}{d}\right)}$$

$$d=T\frac{\log\left(2\right)}{\log\left(\frac{Q_{2}}{Q_{1}}\right)}$$

where V is volume of cell culture media, x is metabolite concentration, A is cell number area under the curve, $N\left(T\right)$ is final cell count, d is doubling time, T is time of experiment, $Q_{1}$ is initial cell number, and $Q_{2}$ is final cell number.

### 2.22 Stable isotope metabolic tracing

Cells in culture were washed with 1X DPBS, trypsinized and resuspended in DMEM (10% FBS, 1% anti-anti). Cells were plated at a density of 52,000 cells/well in 6-well plates. Tracing of labeled nutrients was performed 2 days post-seeding. For tracing of glucose carbons, no-glucose and no-glutamine media was supplemented with 5 mM of ^13^C_6_ d-Glucose (Cambridge Isotope Laboratories, Inc., CLM-1396) and 4 mM unlabeled glutamine. Tracing of glutamine was performed by supplementing no-glucose and no-glutamine media with 4 mM ^13^C_5_ l-glutamine (Cambridge Isotope Laboratories, Inc., CLM-1822-H) and 5 mM unlabeled glucose. Unlabeled controls were maintained on unlabeled low glucose (5 mM) and glutamine (4 mM) media. For metabolite extraction, after 5-h incubation with the isotope tracers, media was removed, and cells were rinsed with 1X DPBS. Then, 500 µL of pre-chilled (on dry ice) analytical-grade 80% methanol (MeOH, Thermo Fisher, AA22909K2): 20% water (Invitrogen, 10977015) (volume/volume) was added to the well. Cell extracts were scraped and transferred to microcentrifuge tubes, and the well was rinsed with another 500 µL of 80% MeOH that was combined with the first round of extract. Samples were vortexed and incubated on dry ice for 15 min, then centrifuged at >15,000 rpm at 4°C for 15 min. Supernatant containing the extracted metabolites was transferred to a new tube and stored at −80°C until further processing. On the day of analysis, samples were dried using a vacuum concentrator (Savant SpeedVac SPD130, Thermo Fisher Scientific), and dried metabolite pellets were resuspended in 60 µL of 60:40 (v/v) acetonitrile: water, vortexed, and centrifuged at >13,000 rpm at 4°C for 15 min. The supernatant was transferred to glass vials with polypropylene inserts for analysis by liquid chromatography-mass spectrometry (LC-MS). The instrument autosampler was maintained at 4°C, and the sample injection volume was 2.5 µL. Samples were analyzed by hydrophilic interaction chromatography coupled to a quadrupole-orbitrap mass spectrometer (Q Exactive, Thermo Fisher Scientific) via electrospray ionization. The liquid chromatography system (Vanquish Flex UHPLC with binary pump VF-P10 and split sampler VF-A10, Thermo Fisher Scientific) used a BEH amide column (ACQUITY Premier BEH Amide VanGuard FIT column, 2.1 mm × 100 mm, 1.7 µm particle size, Waters Corporation #186009508) for separation. The column was held at 35°C, and the flow rate was 300 µL/min with a gradient of solvent A (20 mM ammonium acetate, 20 mM ammonium hydroxide in 95:5 water: acetonitrile (v/v), pH 9.5) and solvent B (acetonitrile). The gradient was 95% B to 40% B from 0 to 9 min, hold 40% B for 2 min, reverse to 95% B in 0.6 min, and hold at 95% B until 20 total min. Samples were directed to the mass spectrometer for 0.25–16 min. The mass spectrometer was operated in negative ion mode with an automatic gain control (AGC) target of 1E6, maximum inject time of 100 min, scan range of 55-825 m/z, and 140,000 resolution. Electrospray ionization source settings included spray voltage of 3 kV and auxiliary gas heater temperature of 350°C with capillary temperature of 325°C. Raw LC-MS data were converted to mzXML file format using “MSConvert” (Chambers et al., 2012; Adusumilli and Mallick, 2017), and data were analyzed using El-MAVEN software (Melamud et al., 2010; Clasquin et al., 2012). Chemical standards for glucose, glutamine, and TCA cycle intermediates were used to validate metabolite identification. Natural ^13^C abundance correction was performed using AccuCor (Su et al., 2017).

## 3 Results

### 3.1 Transformation of primary mouse fibroblasts increases MCU expression, modulates mitochondrial Ca^2+^ uptake, decreases pPDH levels, and promotes respiration during acute Ca^2+^-dependent stimulation

**FIGURE 1:**
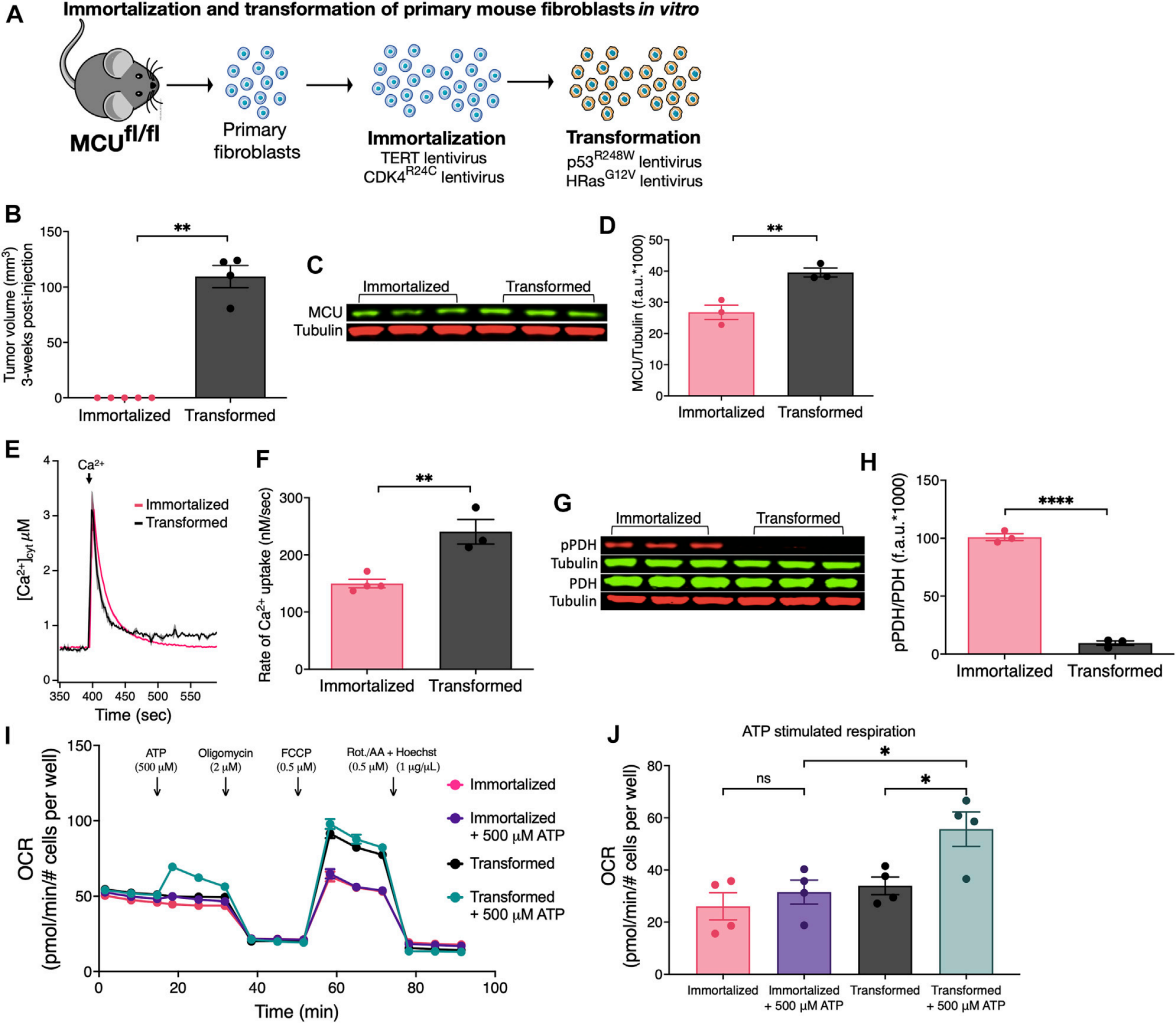
Oncogenic transformation of immortalized primary mouse fibroblasts *in vitro* induces MCU overexpression, faster rates of MCU-mediated Ca^2+^ uptake, decreased pPDH levels, and promotes mitochondrial respiration during acute stimulation. **(A)** Schematic of *in vitro* immortalization and oncogenic transformation of primary mouse fibroblasts. Fibroblasts isolated from 1-week old pups of Mcu^fl/fl^ mice were transduced with the catalytic subunit of telomerase (TERT) and constitutively active CDK4 (CDK4^R24C^) in a sequential manner for immortalization. Cells were either kept in culture as immortalized cells or transduced with dominant-negative p53 (p53^R248W^) and HRas (HRas^G12V^) for oncogenic transformation. **(B)** Tumor xenograft volumes of immortalized and transformed fibroblasts 3 weeks post-injection (mean ± SEM, *n* = 5, **p < 0.01, Student’s t-test). **(C)** Representative immunoblots of endogenous MCU and tubulin in immortalized and transformed fibroblasts. **(D)** MCU levels normalized to tubulin expression detected on same blots. Values expressed as fluorescence units (f.a.u*1000; mean ± SEM, *n* = 3, **p < 0.01). **(E)** Average traces of [Ca^2+^]_cyt_ in suspensions of permeabilized transformed and immortalized fibroblasts (mean ± SEM). **(F)** Mitochondrial Ca^2+^ uptake rates of permeabilized immortalized and transformed fibroblasts (mean ± SEM, *n* = 3, **p < 0.01 by Student’s t-test). **(G)** Representative immunoblots of PDH, pPDH and tubulin in immortalized and transformed fibroblasts. **(H)** Relative protein levels of pPDH and PDH determined by measuring intensities of bands normalized to corresponding tubulin band intensity on same blot (mean ± SEM, *n* = 3, ****p < 0.0001, Student’s t-test). **(I)** Oxygen consumption rates (OCR) of transformed and immortalized fibroblasts (mean ± SEM, *n* = 4) in basal and stimulated conditions. **(J)** Basal and ATP-stimulated respiration in transformed and immortalized fibroblasts (mean ± SEM, *n* = 4, *p < 0.05, ns = non-significant, one-way ANOVA).

To investigate the roles of MCU in tumorigenesis, we developed *in vitro* immortalization and transformation models using a set of transgenes to immortalize and then to transform primary fibroblasts isolated from Mcu^fl/fl^ mice (Figure 1A). Isolated primary fibroblasts were transduced to overexpress TERT, the catalytic subunit of telomerase, and with constitutively-active CDK4 (CDK4^R24C^) (Figure 1A). In combination, these genes efficiently immortalize cells without conferring tumor-forming ability or genomic instability (Sasaki et al., 2009). For malignant transformation, the immortalized fibroblasts were transduced with dominant-negative p53 (p53^R248W^) and HRas (HRas^G12V^) (Figure 1A).

Subcutaneous tumors were produced by injecting immortalized and transformed fibroblasts under the skin of immunocompromised nude (J:Nu) mice. After 3 weeks post-injection, immortalized fibroblasts (*n* = 5) failed to form tumors whereas all injections (*n* = 4) of transformed fibroblasts resulted in tumors (Figure 1B). Thus, transformed fibroblasts have a malignant tumorigenic potential while immortalization serves as syngeneic control for a non-malignant phenotype.

Oncogenic mutations including HRas^G12V^ and p53^R248W^ can induce remodeling of numerous networks to support cancer progression, including mitochondrial biogenesis and turnover (Yao et al., 2019). Mitochondrial HSP60 and Tim23 expression levels were increased ∼1.5-fold in the transformed vs. immortalized fibroblasts (Supplementary Figure S1A), indicating that oncogenic transformation *in vitro* enhanced mitochondrial biomass (Supplementary Figure S1B, C). Similarly, MCU protein expression (Figure 1C) was higher by 1.5-fold in transformed vs. immortalized fibroblasts (Figure 1D). To determine whether increased MCU expression was a transformation-associated process, we quantified MCU in three different primary fibroblast preparations (Fibroblasts WT) before any genetic manipulation (Supplementary Figure S1D). WT fibroblasts expressed lower levels of MCU than transformed ones (Supplementary Figure S1E). These results provide *in vitro* evidence that oncogenic transformation is associated with up-regulation of MCU. To establish the relationship between MCU expression and rates of mitochondrial Ca^2+^ uptake, cells were suspended in an intracellular-like bath solution containing the cell-impermeable Ca^2+^ indicator Fura-FF to monitor bath [Ca^2+^] ([Ca^2+^]_cyt_) and digitonin to solubilize the plasma membrane and expose mitochondria to the bath solution. ER Ca^2+^ uptake and mitochondrial Ca^2+^ extrusion were inhibited by addition of thapsigargin and CGP37157, respectively. Ca^2+^ clearance by mitochondrial Ca^2+^ uptake was measured by changes in [Ca^2+^]_cyt_ after addition of a 3–5 μM bolus of Ca^2+^ to the bath solution. Enhanced MCU expression in transformed fibroblasts was associated with significantly faster rates of mitochondrial Ca^2+^ uptake in permeabilized cells compared with both untransduced WT fibroblasts (Supplementary Figures S1F, G) and immortalized fibroblasts (Figures 1E, F). Enhanced Ca^2+^ uptake observed following transformation was not caused by altered mitochondrial membrane potential (ΔΨ_m_) (not shown, but see Figure 5C). Transformation was also associated with altered [Ca^2+^]_mit_ kinetics in response to ligand-induced intracellular Ca^2+^ release (Supplementary Figure S1H). PDH is activated by dephosphorylation by the mitochondrial matrix Ca^2+^-sensitive PDH phosphatase (PDP) (Denton, 2009). Stimulation of PDH by Ca^2+^ is known to be important for the regulation of mitochondrial metabolism and cancer progression (Denton, 2009; Pan et al., 2013; Luongo et al., 2015; Chakraborty et al., 2017; Anwar et al., 2021). Thus, we examined phosphorylation of PDH by Western blot (Figure 1G). Phospho-PDH (pPDH) was ∼4-fold lower (and almost undetectable) in the transformed cells compared with the immortalized fibroblasts (Figure 1H), suggesting that malignant transformation *in vitro* results in decreased pPDH associated with more efficient ER-to-mitochondria transfer of Ca^2+^ (Cardenas et al., 2010; Baughman et al., 2011; De Stefani et al., 2011; Hall et al., 2014; Tosatto et al., 2016; Ren et al., 2017; Yu et al., 2017; Zhao et al., 2019; Liu et al., 2020). The oxygen consumption rates (OCR) under basal and stimulated conditions were quantified to determine if oncogenic transformation resulted in alterations of mitochondrial respiration (Figure 1I). Surprisingly, cell transformation was associated with only a slight, insignificant enhancement of basal respiration (Supplementary Figure S1I), although uncoupled maximal respiration was significantly increased (Supplementary Figures S1J, K). More importantly, acute stimulation of mitochondrial respiration by ATP activation of metabotropic purinergic receptors promoted a rapid and significant increase of OCR in transformed, but not immortalized fibroblasts (Figure 1J). These data suggest that an increase in transformation-associated MCU expression has no effect on basal mitochondrial respiration, whereas it promotes Ca^2+^-dependent mitochondrial respiration during acute stimulation.

To further establish the relationship between MCU expression and rates of mitochondrial Ca^2+^ uptake, we utilized HEK293T cells with MCU genetically deleted (MCU-KO). In addition, these cells were used to stably express human MCU to create an isogenic HEK293T MCU-rescue line (Supplementary Figure S1L). Rescue of MCU resulted in a ∼2-fold higher expression compared with WT HEK293T levels (Supplementary Figure S1M). Mitochondrial Ca^2+^ uptake in permeabilized cell suspensions was absent in MCU-KO cells whereas it was restored in cells re-expressing MCU (Supplementary Figure S1N). Of note, the rate of Ca^2+^ uptake was ∼2-fold faster in the MCU-rescue cells (Supplementary Figure S1O). There were no differences between ΔΨ_m_ of WT, MCU-KO and MCU-rescue cells (Supplementary Figure S1P), indicating that faster rates in MCU-rescue compared to WT cells were not due to differences in ΔΨ_m_. These results suggest that observed higher levels of MCU expression in tumor cells are associated with enhanced rates of Ca^2+^ uptake in permeabilized cells and altered kinetic responses in intact cells.

### 3.2 MCU is required for tumor growth *in vivo* by promoting cell proliferation

We developed two models to more directly explore the roles of MCU in tumorigenesis *in vivo*. In the first, we generated tumor xenografts in immunodeficient NOD SCID mice injected with HEK293T-WT cells in one flank and either HEK293T MCU-KO or MCU-rescue cells in the other. When examined 4 weeks post-injection, all 3 cell lines had formed tumors. Compared with WT tumors, those formed by MCU-KO cells were considerably smaller (Supplementary Figure S2A): WT tumors had an average volume of ∼900 mm^3^ whereas MCU-KO tumors were ∼100 mm^3^ (Supplementary Figure S2B). Notably, the sizes of tumors generated by MCU-rescue cells were similar to those generated by WT cells (Supplementary Figure S2B). These results suggest that MCU is dispensable for tumor formation, whereas it plays an important role in tumor growth.

**FIGURE 2:**
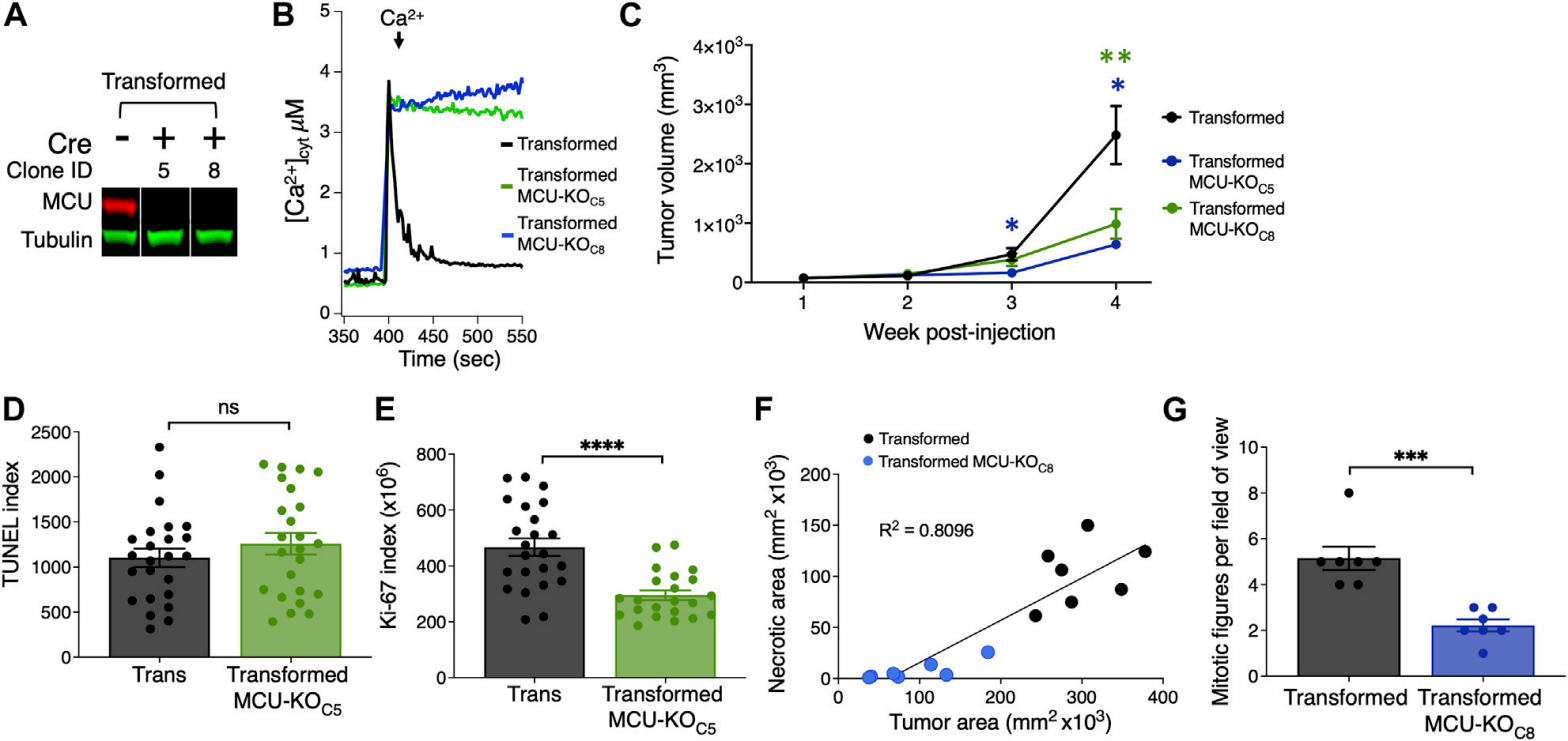
Histological analyses of tumor xenografts reveal that deletion of MCU reduces proliferation and oncogenic potential of primary mouse fibroblasts *in vivo*. **(A)** Representative immunoblots of MCU and tubulin in transformed fibroblasts. Single MCU-KO clones selected from heterogeneous population of transformed fibroblasts expressing mCherry-Cre by FACS. **(B)** Representative traces of [Ca^2+^]_cyt_ in cell suspensions of transformed MCU-KO clones to validate complete elimination of MCU-mediated mitochondrial Ca^2+^ uptake. **(C)** Volumes of tumor xenografts of transformed fibroblasts and MCU-KO fibroblasts during 4 weeks post-injection (mean ± SEM, *n* = 10, *p < 0.05, **p < 0.01, two-way ANOVA). **(D)** Quantification of TUNEL index in tumor xenografts as indicator of DNA damage due to cell death (mean ± SEM, *n* = 3, ns = non-significant, Student’s t-test). **(E)** Quantification of ki-67 index in tumor xenografts as indicator of cell proliferation (mean ± SEM, *n* = 3, ****p < 0.0001, Student’s t-test). **(F)** Necrotic area relative to total tumor area. Necrotic and normal areas quantified manually. **(G)** Quantification of mitotic figures in H&E-stained xenograft sections. For each tumor, 10 different fields of view were examined at high magnification (×40) (mean ± SEM, *n* = 7, ***p < 0.001, Student’s t-test).

In the second model, we induced tumor xenografts in immunocompromised nude (J:Nu) mice using transformed fibroblasts. We genetically eliminated MCU (MCU-KO) by expressing mCherry-Cre recombinase in the Mcu^fl/fl^-transformed fibroblasts and selecting mCherry-positive cells. Genetic deletion of MCU in clonal lines was validated by Western blot (Figure 2A) and by mitochondrial Ca^2+^ uptake assays (Figure 2B). Both cell lines formed tumors (Supplementary Figure S2C), but MCU-KO tumors were significantly (>60%) smaller (Figure 2C).

To explore the mechanisms underlying reduced tumor size associated with lack of MCU, we quantified cell death and the proliferation index of fibroblast tumor xenografts. Paraffin-embedded tumor slices were stained with DAPI, immunolabeled for Ki-67 as a marker of cell proliferation, and TUNEL (terminal deoxynucleotidyl transferase-mediated dUTP Nick-end labeled)-stained as a marker of apoptotic cell death (Supplementary Figure S2D). Notably, cell death was not enhanced in tumors formed by MCU-KO transformed fibroblasts compared with those formed by MCU-expressing transformed fibroblasts (Figure 2D). In contrast, the proliferation index of MCU-KO tumors was markedly lower than in the tumors of transformed fibroblasts (Figure 2E). To validate this, we analyzed small tumor-tissue sections and counted individual Ki-67^+^ cells (Supplementary Figure S2E). The proliferation potential of transformed tumor fibroblasts was strikingly decreased by elimination of MCU (Supplementary Figure S2F). These results suggest that MCU-KO does not eliminate the tumorigenic potential of transformed fibroblasts, whereas it markedly slows tumor growth primarily by strongly reducing cell proliferation with lesser enhancement of cell death.

To further understand the mechanism by which deletion of MCU impedes tumor growth, we performed histological analyses of tumors (Supplementary Figure S2G), examining characteristics associated with tumor growth patterns, including amount of normal and necrotic tumor tissue (Supplementary Figure S2H) and mitotic activity (Supplementary Figure S2I). Necrosis in tumors often indicates aggressiveness associated with high cell density due to rapid cell division with low availability of nutrients and anoxic conditions. In agreement, the smaller tumors formed by MCU-KO transformed fibroblasts were associated with significantly reduced size of necrotic areas (Figure 2F). The number of mitotic figures was considerably lower in MCU-KO tumors (Figure 2G), in agreement with the Ki-67 analysis. Some cells with morphological characteristics associated with senescence, namely markedly large cell size and/or polyploidy, were recognized as giant multinucleated cells (Supplementary Figure S2J). Of interest, a substantially higher number of multinucleated giant cells was observed in MCU-KO tumors (Supplementary Figure S2K).

### 3.3 Deletion of MCU reduces cell proliferation of primary mouse fibroblasts

The results from the *in vivo* transformed fibroblast tumor model suggests that MCU is required for cell proliferation to support tumorigenesis. We undertook a series of *in vitro* experiments to evaluate the effects of MCU depletion on cell proliferation, as well as other cellular phenotypes that drive cancer cell malignancy, including inhibition of cell death, cell division, sphere formation and matrix invasion.

**FIGURE 3:**
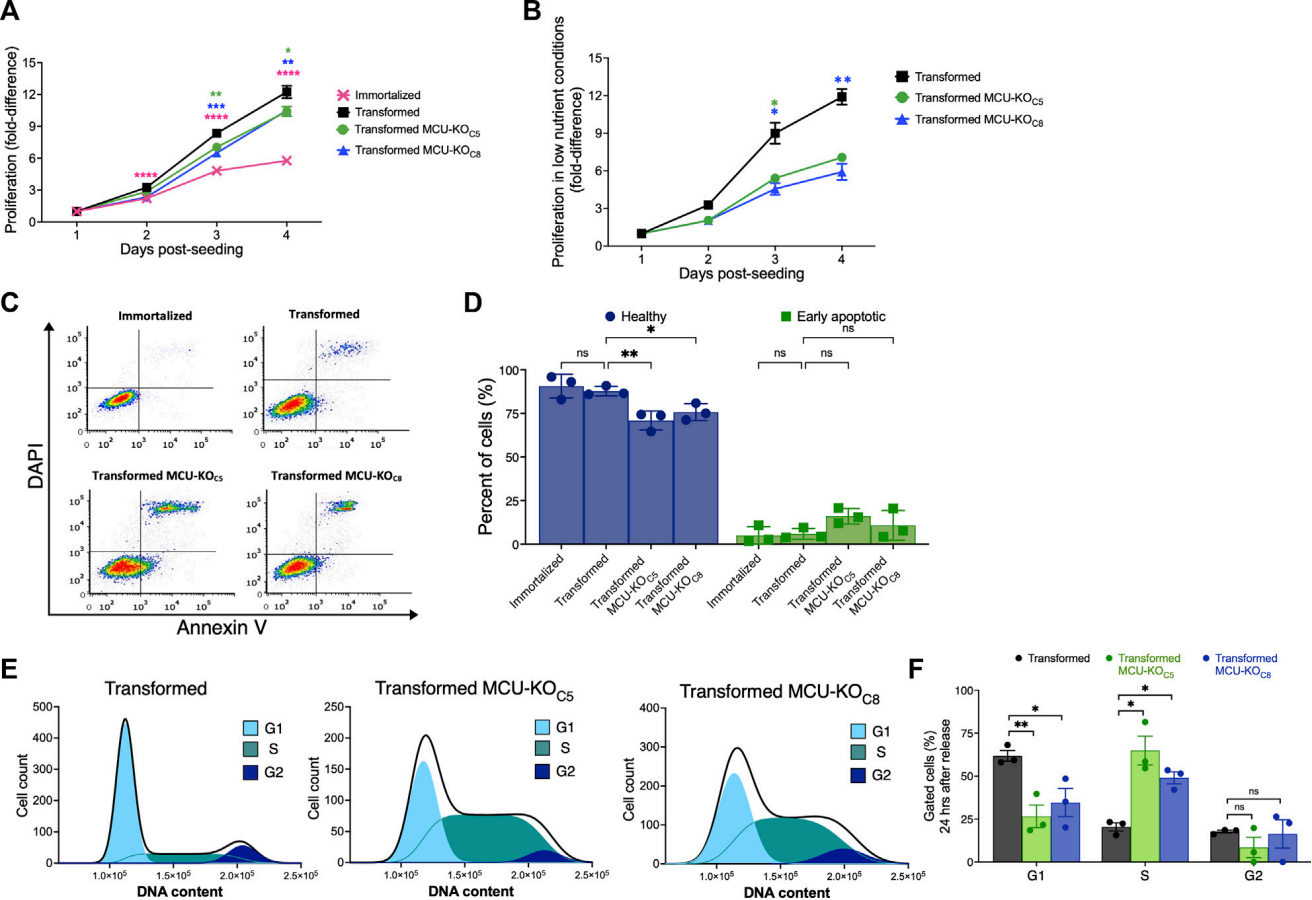
Deletion of MCU reduces proliferative potential, invasion ability, and delays cell-cycle progression of transformed mouse fibroblasts *in vitro*. **(A)** Proliferation of immortalized and transformed fibroblasts. Each data point represents three biological replicates in triplicate. Fold-difference represents number of cells normalized to cell count at day 1 post-seeding (mean ± SEM, *n* = 3, *p < 0.05, **p < 0.01, ****p < 0.0001, two-way ANOVA compared to transformed cells). **(B)** Proliferation of transformed fibroblasts in low-nutrient conditions. Each data point represents four biological replicates in triplicate. Fold-difference represents number of cells normalized to day 1 post-seeding. (mean ± SEM, *n* = 4, *p < 0.05, **p < 0.01, two-way ANOVA compared to transformed cells). **(C)** Representative FACS plots of Annexin-V/DAPI-stained cells. **(D)** Percent (%) healthy cells (lower-left quadrant, purple) and early apoptotic cells (lower-right quadrant, green) of each individual FACS plot significantly different in (C) (mean ± SEM, *n* = 3, *p < 0.05, **p < 0.01, ns = non-significant, two-way ANOVA compared to transformed cells). **(E)** Representative histograms of DNA-content distribution of transformed fibroblasts collected after 24 h of release from lovastatin synchronization. Samples were fixed, stained with DAPI, and FACS sorted for quantification. **(F)** Quantification of percent (%) of gated cells in G1, S, and G2 after 24 h release from lovastatin synchronization for transformed fibroblasts (black bars), MCU-KO clone 5 (green bars), and MCU-KO clone 8 (blue bars) (mean ± SEM, *n* = 3, *p < 0.05, **p < 0.01, ns = non-significant, two-way ANOVA compared to transformed cells).

The transformed fibroblasts proliferated significantly faster than the immortalized fibroblasts, as expected (Figure 3A). Notably, proliferation of the transformed fibroblasts was only moderately reduced by genetic deletion of MCU in both clonal MCU-KO lines examined (Figure 3A). MCU-KO mice have no apparent physiological phenotypes until they are physically stressed (Kwong et al., 2015; Luongo et al., 2015). Therefore, we examined proliferation of transformed fibroblasts under low-nutrient conditions, a stress imposed in the tumor microenvironment. Fibroblasts were initially seeded in high glucose (25 mM) and glutamine (4.5 mM) media, and after 24 h the media was changed to one with low glucose (4.5 mM) and glutamine (0.75 mM) that was sufficient to allow fibroblasts to proliferate and remain viable for a period of 4 days post-seeding. Under these conditions, genetic deletion of MCU much more strongly decreased proliferation of transformed fibroblasts (Figure 3B). To confirm the requirement of MCU expression for optimal cell proliferation, we generated MCU-rescue cell lines, which were validated by Western Blot (Supplementary Figures S3A, B) and by Ca^2+^ uptake assays (Supplementary Figures S3C, D). Proliferation was enhanced in both rescue cell lines in both nutrient-rich (Supplementary Figure S3E) as well as in nutrient-poor (Supplementary Figure S3F) conditions, confirming that MCU expression correlates with cell-proliferative capacity and ruling out the possibility that decreased proliferation of MCU-KO cells was due to non-specific effects of Cre expression.

To determine the mechanisms of reduced proliferation of transformed cells lacking MCU, we examined cell death and cell-cycle progression. Apoptotic and non-apoptotic cell death were assessed by annexin-V/DAPI staining and fluorescence-activated cell sorting (FACS) (Figure 3C). Populations of live, dead, apoptotic, and necrotic cells were established based on viable and 1 μM staurosporine-treated controls (Supplementary Figure S3G). There were no differences in the percent of either non-viable (early/late apoptotic and necrotic cells) or healthy cells between the immortalized and transformed populations (Supplementary Figure S3H). Genetic deletion of MCU diminished the percentage of healthy cells and moderately increased the number of early-apoptotic cells (Figure 3D). Thus, the major effect of MCU-KO in transformed cells was to decrease cell proliferation. Notably, these *in vitro* results are highly consistent with the conclusions reached in the *in vivo* model.

To understand the basis for reduced proliferation observed *in vivo* and *in vitro*, we examined the effects of MCU deletion on cell-cycle progression. Fibroblasts were synchronized in the G1 phase using lovastatin (Keyomarsi et al., 1991), and then examined by DAPI-FACS 24 h after release from synchronization. Unsynchronized and lovastatin-synchronized samples provided controls (Supplementary Figure S3I). At 24 h after release from synchronization, a significant fraction of the transformed cells progressed through mitosis into the G2 phase of the cell cycle (Figures 3E, F). In contrast, both MCU-KO clonal cell lines contained a significantly lower percent of cells in G1 phase and a much higher percentage in S phase (Figures 3E, F). Accumulation of MCU-KO cells in the S phase suggests that MCU is important for cell proliferation by promoting progression through the cell cycle (Cardenas et al., 2016; Koval et al., 2019; Zhao and Pan, 2021).

### 3.4 Deletion of MCU reduces cancer-associated phenotypes of transformed mouse fibroblasts

**FIGURE 4:**
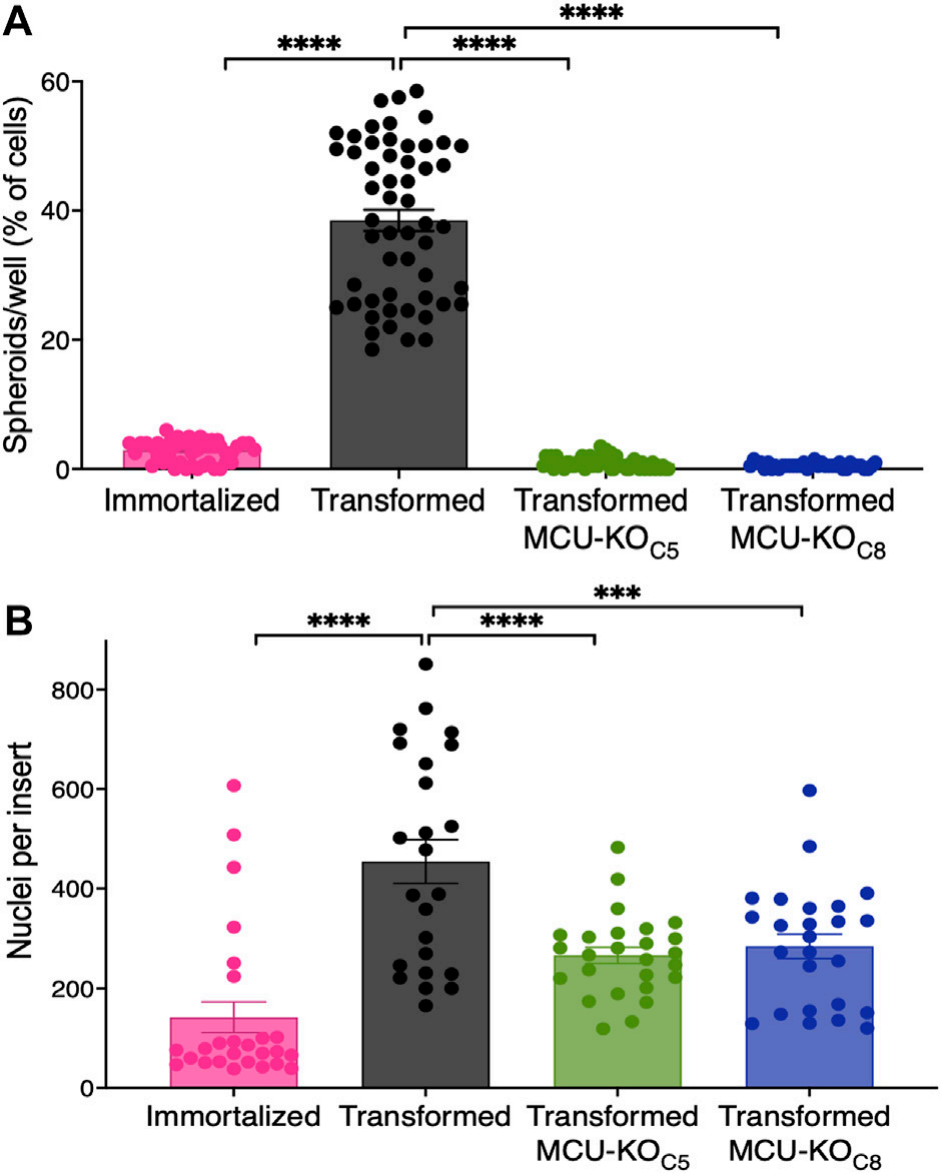
MCU deletion inhibits sphere formation capacity and reduces cell invasion *in vitro*. **(A)** Number of spheroids formed as a percent of number of cells seeded. 200 cells were plated in each well of low adhesion plates and spheroids were counted 7 days later (mean ± SEM, *n* = 4, ****p < 0.0001, one-way ANOVA compared to transformed cells). **(B)** Transwell invasion assay. 15,000 cells were seeded atop Matrigel-coated Transwells. After 24 h, membranes were fixed and stained with Hoechst 33342 to quantify number of invading cells. Three different fields of view of each well were imaged and quantified (mean ± SEM, *n* = 3, ****p < 0.0001, one-way ANOVA compared to transformed cells).

The ability to form clonally-derived spheres on non-adherent substrates is a stem cell-like capacity related to metastatic tumor initiation and progression (Uchida et al., 2010). In a sphere-formation assay, the transformed fibroblasts, but not the immortalized cells, readily formed spheres (>30 spheres per well) (Figure 4A), consistent with their differential abilities to form tumors *in vivo* (Figure 1B). In contrast, MCU-KO transformed fibroblasts formed <5 spheres per well (Figure 4A). These results suggest that MCU plays an important role in both cell proliferation as well as the capacity to self-renew.

To explore the role of MCU in cell chemotaxis and extracellular matrix invasion, processes associated with cancer metastasis in which Ca^2+^ signaling has been proposed to play a role (Tang et al., 2015; Tosatto et al., 2016), we employed a transwell-invasion assay. Cells were seeded atop a porous Matrigel-coated membrane and examined after 24 h (Supplementary Figure S4A). The number of transformed fibroblasts that penetrated through the coated membrane was ∼3-fold greater than the number of immortalized fibroblasts that traversed it (Figure 4B). Importantly, genetic deletion of MCU in transformed fibroblasts markedly reduced the number of invading cells by ∼50% (Figure 4B).

Together, these results validate our transformed fibroblasts as a model that recapitulates many phenotypes associated with malignancies, including enhanced proliferation, ability to form clonally-derived spheres, and cell migration and tissue invasion. Importantly, elimination of MCU strongly suppressed these *in vitro* phenotypes, suggesting that it could play an important role in carcinogenesis, as observed in our *in vivo* models.

### 3.5 Deletion of MCU in transformed primary mouse fibroblasts increases glycolysis

**FIGURE 5:**
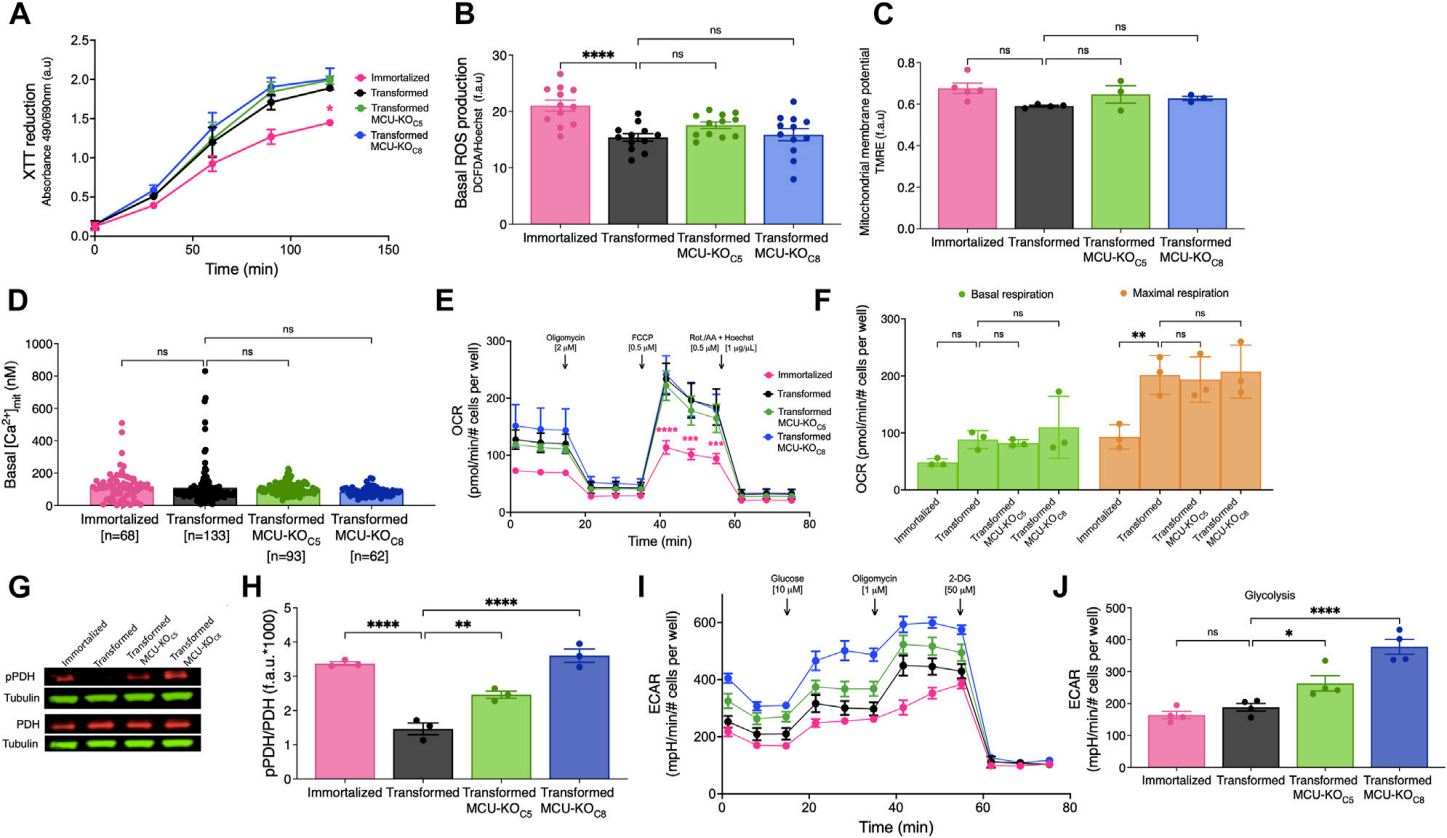
Cell physiological effects of MCU deletion in transformed primary mouse fibroblasts *in vitro*. **(A)** Metabolic activity measured by XTT reduction (mean ± SEM, *n* = 3, *p < 0.05, two-way ANOVA compared to that of transformed fibroblasts). **(B)** Basal reactive oxygen species (ROS) production measured over 45 min by DCFDA fluorescence normalized to Hoechst 33342 (mean ± SEM, *n* = 3, ****p < 0.0001, ns = non-significant, one-way). **(C)** Normalized ΔΨ_m_ measured by TMRE fluorescence (f.a.u) in permeabilized cell suspension (mean ± SEM, *n* = 3, ns = non-significant, one-way ANOVA). **(D)** Resting mitochondrial matrix [Ca^2+^] ([Ca^2+^]_mit_) measured with the indicator CEPIA2mt. Each cell was individually analyzed (n = number of cells; mean ± SEM, ns = non-significant, one-way ANOVA). **(E)** OCR of transformed and immortalized fibroblasts. Mean values compared to those of transformed fibroblasts (mean ± SEM, *n* = 3, ***p < 0.001, ****p < 0.0001, two-way ANOVA compared to transformed fibroblasts). **(F)** Basal (green bars) and maximal (orange bars) respiration of transformed and immortalized fibroblasts (mean ± SEM, *n* = 3, **p < 0.005, ns = non-significant, two-way ANOVA). **(G)** Representative immunoblots of PDH, pPDH and tubulin in transformed and immortalized fibroblasts. **(H)** Relative protein levels of pPDH and PDH determined by measuring intensities of bands normalized to corresponding tubulin band intensity on same blot (mean ± SEM, *n* = 3, **p < 0.01, ****p < 0.0001, one-way ANOVA). **(I)** Glycolytic extracellular acidification rates (ECAR) of immortalized and transformed fibroblasts (mean ± SEM, *n* = 4). **(J)** Basal glycolytic ECAR of immortalized and transformed fibroblasts. For statistical analysis, mean values were compared to that of transformed fibroblasts (mean ± SEM, *n* = 4, *p < 0.05, ****p < 0.0001, ns = non-significant, one-way ANOVA).

To investigate mechanisms by which elimination of MCU affects cancer-progression phenotypes, we examined several mitochondrial functions. First, we examined total dehydrogenase activity using the XTT assay. Oncogenic transformation significantly increased dehydrogenase activity, but genetic deletion of MCU was without effect (Figure 5A). Similarly, ROS production was decreased after transformation but was not changed by deletion of MCU (Figure 5B). ΔΨ_m_ was not significantly different after transformation or in MCU-KO fibroblasts (Figure 5C). In addition, mitochondrial matrix [Ca^2+^] ([Ca^2+^]_mit_) was also not different after transformation or in MCU-KO cells (Figure 5D). Together these findings indicate that reduced malignancy of MCU-KO transformed fibroblasts is not associated with reduced mitochondrial function.

To further explore the role of MCU in cellular bioenergetics, we quantified OCRs (Figure 5E). Unexpectedly, neither basal nor maximal respiration of transformed fibroblasts was affected by genetic deletion (Figure 5F) or rescue (Supplementary Figures S5A, B) of MCU. Spare respiratory capacity, proton leak, coupling efficiency, ATP production, and non-mitochondrial OCR were also not altered by transformation, MCU-KO or MCU-rescue (data not shown). Nevertheless, diminished phosphorylation of PDH associated with transformation (Figure 2G) was strongly suppressed by genetic deletion of MCU (Figures 5G, H) and enhanced by MCU-rescue (Supplementary Figures S5C, D). Although genetic deletion of MCU did not affect OCR, it significantly increased glycolysis (Figures 5I, J) and glycolytic capacity (Supplementary Figure S5E). The glycolytic reserve of fibroblasts was increased after transformation and remained unchanged after MCU-KO (Supplementary Figure S5F). No changes were observed in non-glycolytic acidification rates after transformation or deletion of MCU (data not shown). Thus, the major effects of MCU deletion on cellular bioenergetics *in vitro* was suppression of PDH phosphorylation and upregulation of glycolysis, while mitochondrial respiration remained unaffected.

### 3.6 Deletion of MCU in transformed primary mouse fibroblasts alters cellular metabolism

**FIGURE 6:**
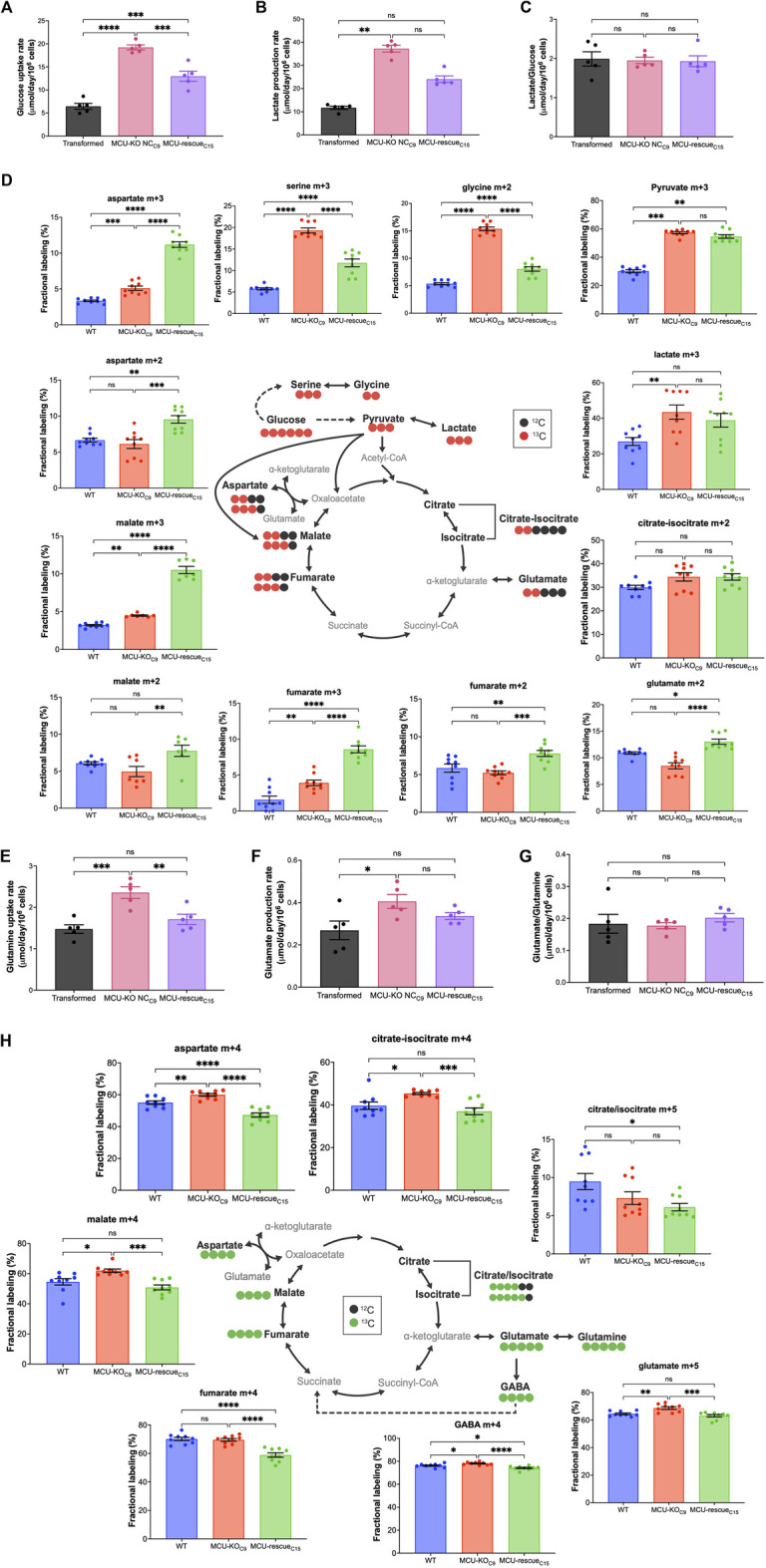
Deletion of MCU alters glucose and glutamine metabolism. (**A**) Glucose-uptake rates of transformed fibroblasts (mean ± SEM, *n* = 5, ***p < 0.001, ****p < 0.0001, one-way ANOVA). (**B**) Lactate-production rates of transformed fibroblasts (mean ± SEM, *n* = 5, **p < 0.01, ns = non-significant, one-way ANOVA). (**C**) Lactate-production rates normalized to glucose-uptake rates (mean ± SEM, *n* = 5, ns = non-significant, one-way ANOVA). (**D**) Schematic representation of metabolic routes, labeling pattern of ^13^C_6_ d-Glucose-derived metabolites, and fractional labeling of isotopologues of interest (mean ± SEM, *n* = 8, ns = non-significant, *p < 0.05, **p < 0.01, ***p < 0.001, ****p < 0.0001, one-way ANOVA). Red circles represent labeling with ^13^C and black circles represent naturally-occurring ^12^C. Isotopologues of interest are represented by mass (m) and total number of ^13^C. (**E**) Glutamine-uptake rates of transformed fibroblasts (mean ± SEM, *n* = 5, ns = non-significant, **p < 0.01, ***p < 0.001, one-way ANOVA). (**F**) Glutamate-production rates of transformed fibroblasts (mean ± SEM, *n* = 5, ns = non-significant, *p < 0.05, one-way ANOVA). (**G**) Glutamate-production rates normalized to glutamine-uptake rates (mean ± SEM, *n* = 5, ns = non-significant, one-way ANOVA). (**H**) Schematic representation of metabolic routes, labeling pattern of ^13^C_5_ l-Glutamine-derived metabolites, and fractional labeling of isotopologues of interest (mean ± SEM, *n* = 8, ns = non-significant, *p < 0.05, **p < 0.01, ***p < 0.001, ****p < 0.0001, one-way ANOVA). Green circles represent labeling with ^13^C and black circles represent naturally occurring ^12^C.

Altered metabolism is a fundamental characteristic of cancer cells (Hanahan and Weinberg, 2011). Cancerous cells metabolize large quantities of glucose into lactate to support rapid energy and biomass production for cellular growth and proliferation (Hatzivassiliou et al., 2005; DeBerardinis et al., 2008a; Deberardinis et al., 2008b; Lunt and Vander Heiden, 2011; Ganapathy-Kanniappan and Geschwind, 2013; Liberti and Locasale, 2016). Indeed, the observed increase in ECAR in MCU-KO cells was associated with increased glucose uptake (Figure 6A) and lactate production (Figure 6B), effects that were attenuated by MCU re-expression (Figures 6A, B). When the rate of lactate production was normalized to glucose uptake rate, there were no significant difference between cell lines (Figure 6C), suggesting that glucose is largely metabolized to lactate through glycolysis in MCU-KO cells. To determine if increased glucose consumption in MCU-KO cells alters cellular metabolism, we performed metabolic tracing with ^13^C_6_ d-Glucose (Figure 6D). Fractional labeling all metabolites included in our analysis is shown in Supplementary Table S1. The network depicted in Figure 6D represents metabolic routes, labeling pattern of ^13^C_6_ d-Glucose-derived metabolites, and fractional labeling of isotopologues of interest. Glucose entry into the TCA cycle via acetyl-CoA and PDH leads to labeling of TCA cycle intermediates with two ^13^C, represented by m+2. Alternatively, entry of glucose-derived ^13^C into the TCA cycle via pyruvate carboxylation by pyruvate carboxylase (PC) or malic enzyme (ME) results in the incorporation of three ^13^C in TCA-cycle intermediates, represented by m+3. Labeling of lactate in aerobic glycolysis (m+3) was significantly increased in MCU-KO as compared to transformed cells (Figure 6D). Notably, labeling of m+3 serine and m+2 glycine was also increased in transformed MCU-KO cells. This phenotype was reversed by MCU-rescue (Figure 6D), suggesting that deletion of MCU promotes the diversion of glucose-derived carbons into biosynthetic pathways, such as for purine biosynthesis (Vanhove et al., 2019). In all transformed cell lines, m+2 citrate was ∼30% of the labeling from glucose, while labeling of m+2 fumarate and malate was ∼5–10%, suggesting exit of citrate from the TCA cycle to fuel biosynthetic reactions such fatty-acid synthesis. Interestingly, m+2 glutamate, fumarate, malate, and aspartate levels were not different between transformed and MCU-KO cells, although rescue of MCU enhanced their labeling beyond those observed in the wild-type transformed cells (Figure 6D). This result is consistent with the observed lack of effect of MCU deletion on basal OCR. Notably, elevated levels of m+3 fumarate, malate, and aspartate indicate that entry of pyruvate through alternative pathways, likely PC, was significantly enhanced by MCU-KO (Figure 6D). These results suggest a striking metabolic adaptation in MCU-KO cells that promotes serine biosynthesis and TCA cycle activity.

Many cancer cells shift their substrate preference to fuel flux through the TCA cycle (Reitzer et al., 1979). Glutamine provides another key carbon source for the TCA cycle through anaplerosis (DeBerardinis et al., 2007; Wise et al., 2008). We therefore evaluated the contribution of glutamine to the TCA cycle. The schematic in Figure 6H represents the labeling pattern of ^13^C_5_ l-Glutamine-derived TCA-cycle intermediates, γ-aminobutyric acid (GABA), and fractional labeling of isotopologues of interest. Isotopic labeling with ^13^C is represented by green circles, while naturally-occurring ^12^C is depicted as black circles. Glutamine anaplerosis by oxidation of α -KG results in a m+4 mass increase of TCA-cycle intermediates. On the other hand, partial reverse flow of the TCA cycle for reductive carboxylation of α -KG by enzymatic activity of Ca^2+^-independent IDH2 leads to formation of m+5 citrate-isocitrate. Fractional labeling of all metabolites included in our analysis is shown in Supplementary Table S2. Genetic deletion of MCU was associated with an increased glutamine uptake (Figure 6E) and its conversion to glutamate (Figure 6F). With glutamate production normalized to glutamine uptake rate, there was no difference between cell lines (Figure 6G), suggesting that MCU deletion was primarily associated with increased nutrient uptake rather than involvement of alternative glutamine metabolic pathways. In agreement, ^13^C_5_-glutamine tracing revealed increased labeling of m+5 glutamate, m+4 aspartate, m+4 citrate-isocitrate, and m+4 malate in MCU-KO vs. WT transformed cells, which was reversed by MCU re-expression (Figure 6H). We also found a significant increase in the diversion of glutamine-derived carbons into the GABA shunt in MCU-KO cells and significant decrease with MCU-rescue (Hoang et al., 2021). The GABA shunt can serve as a reservoir for TCA cycle anaplerosis by promoting entry of glutamine-derived carbons through succinate and by passing α -KGDH (Figure 6H). Taken together, these data suggest that in the absence of MCU, glucose metabolism via glycolysis is enhanced, and cells rely more on glutamine to maintain TCA-cycle integrity.

### 3.7 Deletion of MCU in transformed primary mouse fibroblasts alters agonist-induced cytoplasmic Ca^2+^ signals

**FIGURE 7:**
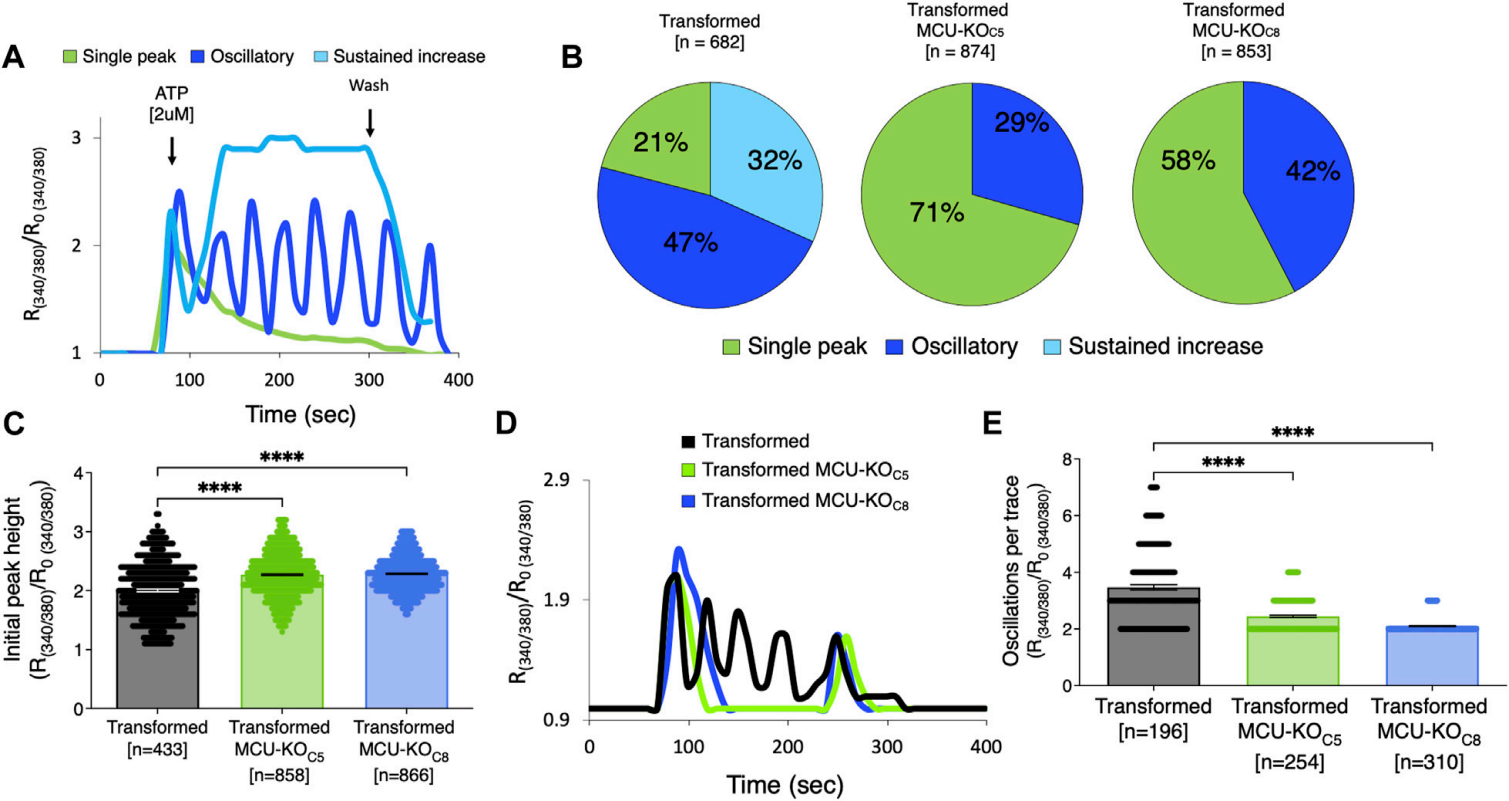
Deletion of MCU alters cytosolic Ca^2+^ transients induced by activation of metabotropic purinergic receptors by ATP. **(A)** Representative traces of relative cytoplasmic [Ca^2+^] measured with Fura-2 and represented as R/R_0_ for normalization, where R is the ratio of Fura-2 fluorescence at 340:380 nm excitation. ATP-induced responses in transformed fibroblasts classified as single spike (green trace), oscillations (blue trace), or sustained rise of [Ca^2+^]_cyt_ (light blue trace). **(B)** Percent (%) of ATP-induced responses classified as single spike (green), oscillatory (blue), and sustained rise of [Ca^2+^]_cyt_ (light blue) in transformed fibroblasts and MCU-KO fibroblasts. Each cell was individually analyzed (*n* = number of cells). **(C)** Height of initial peak R/R_0_ after addition of ATP (*n* = number of cells; mean ± SEM, ****p < 0.0001, one-way ANOVA). (**D**) Representative traces of oscillatory responses induced by ATP in transformed (black trace), MCU-KO clone 5 (green trace), and MCU-KO clone 8 (blue trace) fibroblasts. (**E**) Number of oscillations over 300 s in responses classified as oscillatory (n = number of cells; mean ± SEM, ****p < 0.0001, one-way ANOVA).

Because mitochondria can play an important role in buffering changes of [Ca^2+^]_cyt_, and alterations in [Ca^2+^]_cyt_ may regulate cell-biological functions of tumor cells, we also examined InsP_3_R-mediated Ca^2+^ signaling in transformed fibroblasts and two clones with MCU deleted. Exposure of cells to ATP elicited three types of responses: a single transient spike, oscillations, and a sustained rise (Figure 7A). In transformed fibroblasts, 32% of cells responded with a sustained elevation of [Ca^2+^]_cyt_, 47% displayed [Ca^2+^]_cyt_ oscillations, and 21% responded with a single [Ca^2+^]_cyt_ spike (Figure 7B). In contrast, a sustained elevation was rarely observed in transformed fibroblasts lacking MCU, with cells responding with either single spikes (∼41%) or oscillations (∼56%) (Figure 7B). The amplitude of the first [Ca^2+^]_cyt_ peak was elevated in the cells lacking MCU (Figure 7C). Among the oscillatory responses (Figure 7D), those in the cells with MCU deleted were of lower frequency compared with those of transformed fibroblasts (Figure 7E). Taken together, these results suggest that [Ca^2+^]_cyt_ signaling is altered in transformed cells by deletion of MCU.

## 4 Discussion

In the present study, we examined the effects of genetic deletion of the MCU pore-forming subunit of the mitochondrial Ca^2+^ uniporter on tumorigenesis *in vivo* and *in vitro* models. We identified a transformation-associated increase of MCU expression that was associated with enhanced Ca^2+^ uptake and cell proliferation. Genetic deletion of MCU dramatically reduced tumor burden *in vivo*, due to a strong reduction in the proliferative capacity of cells *in vivo* and *in vitro*, particularly under conditions of nutrient stress. Although loss of MCU was associated with enhanced phosphorylation of PDH, mitochondrial respiration was unaffected and pyruvate was diverted to lactate and PDH-independent anaplerosis. In addition, anaplerotic glutamine metabolism was enhanced and agonist-induced Ca^2+^ signaling was altered. Reduced proliferation, delayed cell-cycle progression, increased glycolytic and glutamine metabolism, and a dependence on glucose and glutamine for rapid proliferation suggest that genetic deletion of MCU creates an underlying metabolic defect that strongly inhibits tumorigenesis.

We observed a significant increase in MCU expression as a consequence of cell transformation. To our knowledge, this is the first demonstration of MCU upregulation as a direct consequence of malignant transformation and acquisition of an oncogenic potential. Increased MCU expression resulted in enhanced mitochondrial Ca^2+^ uptake in permeabilized cells *in vitro*, in agreement with previous studies (Baughman et al., 2011; De Stefani et al., 2011; Chaudhuri et al., 2013), as well as altered [Ca^2+^ ]_mit_ kinetics in intact cells. Hepatic (Ren et al., 2017), breast (Tosatto et al., 2016), and colorectal (Liu et al., 2020) cancers display high expression levels of MCU that positively correlate with tumor size, metastasis, and poor survival prognosis of patients (Curry et al., 2013; Hall et al., 2014). Of note, a worse prognosis in acute myeloid leukemia (Shi et al., 2015), increased proliferation of hepatocellular carcinomas (Guerra et al., 2019), and stronger migratory capacity of breast cancers (Mound et al., 2017) are associated with overexpression of InsP_3_Rs. These observations suggest that enhanced ER-to-mitochondria Ca^2+^ transfer may be a feature associated with many types of cancer. Transformation also facilitated a rapid stimulation of respiration in response to an acute cytoplasmic Ca^2+^ signal, which may be crucial for energy regulation to sustain increased bioenergetic demand during cancer progression (Cardenas et al., 2010; Koval et al., 2019; Bustos et al., 2021; Herst et al., 2022).

To determine the role of MCU in tumor progression, we performed subcutaneous xenograft experiments with immortalized and transformed fibroblasts and isogenic HEK293T cells. Cell death was only minimally enhanced by MCU deletion *in vivo*. The absence of significant cell death contrasts with results from our previous *in vitro* studies (Cardenas et al., 2010; Cardenas et al., 2016). A distinction between those studies and the present one is that the former examined the acute effects of MCU knockdown whereas the present studies used cells with MCU stably deleted. Similarly, acute knockdown of InsP_3_R caused cancer cell death (Cardenas et al., 2010; Cardenas et al., 2016), whereas stable deletion of all InsP_3_R was associated with a significantly repressed proliferative capacity (Young et al., 2022). Compensatory mechanisms have been proposed to account for the lack of overt physiological consequences of MCU-KO in mice of mixed genetic backgrounds (Garbincius et al., 2020). It is likely that metabolic rewiring, such as enhanced nutrient metabolism and switch in TCA substrate preference from glucose to glutamine, as shown here, is a compensatory mechanism that protect against cell death in stable MCU-KO cell lines used in both our *in vivo* and *in vitro* studies.

In agreement with Tosatto et al. (2016), MCU deletion did not prevent tumor formation but it strongly inhibited tumor growth. Genetic deletion of MCU delayed tumor growth primarily by decreasing cell proliferation that resulted in a smaller tumor size, lower Ki-67 index and reduced number of mitotic cells. In addition, lack of MCU was associated with the appearance of giant multinucleated cells, which may reflect quiescent cells that contribute to cancer dormancy. Senescence has been previously associated with reduced transfer of Ca^2+^ from the ER-to-mitochondria (Huang et al., 2000). Furthermore, maintenance of quiescence and escape of hematopoietic stem cells (HSC) from quiescence requires mitochondrial Ca^2+^ uptake (Resende et al., 2010; Umemoto et al., 2018; Ahumada-Castro et al., 2021; Paliwal et al., 2021). Inhibition of cell proliferation by MCU-KO is consistent with anti-proliferative effects of MCU suppression in cancer cells (Cardenas et al., 2016; Ren et al., 2017; Li et al., 2020; Liu et al., 2020; Wang et al., 2020; Miao et al., 2021; Wu et al., 2021; Zhao et al., 2021), although this has not been universally observed (Curry et al., 2013; Hall et al., 2014; Tosatto et al., 2016; Young et al., 2022). Despite slower tumor growth in xenografts, proliferation *in vitro* of several breast cancer cell lines were independent of MCU (Tosatto et al., 2016), and it has been reported that stable deletion of MCU in HEK293T and HeLa cell lines resulted in enhanced cell proliferation (Young et al., 2022). It is likely that the cell-physiological implications of MCU deletion are cell-type and context-dependent with phenotypes influenced by endogenous metabolic programs (Jose et al., 2011; Pan et al., 2013; Harrington and Murphy, 2015; Luongo et al., 2015; Gui et al., 2016; Marchi et al., 2019; Sullivan and Vander Heiden, 2019). Importantly, the highly-similar effects of MCU deletion in isogenic fibroblasts on cell proliferation *in vivo* and *in vitro* in the present study strongly suggests that MCU is essential for cancer cell proliferation, particularly in tumorigenesis.

We found that inhibitory phosphorylation of PDH, the pyruvate gateway to the TCA cycle, is increased in transformed fibroblasts by genetic deletion of MCU and strongly suppressed by MCU rescue. Increased PDH phosphorylation has been consistently observed in response to MCU deletion (Cardenas et al., 2010; Pan et al., 2013; Luongo et al., 2015; Young et al., 2022). The changes we observed are as expected if constitutive mitochondrial Ca^2+^ influx through MCU drives pyruvate dehydrogenase phosphatase (PDP) activity. It is interesting to note that stimulation of Ca^2+^-sensitive dehydrogenases is observed at >500 nM [Ca^2+^]_mit_ (Denton and McCormack, 1980), whereas we observed that basal [Ca^2+^]_mit_ is ∼100 nM in either the presence or absence of MCU. Correlation of the phospho-status of PDH with MCU expression may suggest that PDP is exposed to local high [Ca^2+^]_mit_ that was not resolved in our study. Despite alterations of phospho-PDH, neither basal or maximal respiration were affected by genetic deletion or rescue of MCU in our transformed fibroblasts. Discrepancy between the phospho-status of PDH and mitochondrial respiration suggests that phosphorylation status of PDH in MCU-KO cells may not always faithfully reflect PDH activity. In agreement, our isotope tracing results suggest that flux of carbons from glucose through PDH is not substantially affected by MCU deletion. Glucose-derived m+2 labeled glutamate, fumarate, malate, and aspartate were not different between transformed wild-type and MCU-KO cells, although rescue of MCU enhanced their labeling beyond those observed in the wild-type transformed cells. The latter observation may suggest that our MCU-KO cells have employed an adaptive metabolic program that promotes TCA-cycle activity independent of MCU. Such an adaptation likely contributes to the observed lack of difference in the OCR between transformed cells with or without MCU. In agreement with our results, strongly enhanced PDH phosphorylation in stable MCU-KO HEK and HeLa cells was not associated with altered glucose-derived carbon flux through PDH or TCA-cycle Ca^2+^-sensitive IDH3 and α-KGDH (Young et al., 2022). PDH is regulated by other factors including elevated NAD^+^/NADH levels observed in MCU-KO cells (Bowker-Kinley et al., 1998; Roche et al., 2003; Patel and Korotchkina, 2006). Elevated pyruvate in chronic MCU-deleted cells could promote sufficient flux through PDH to fuel the TCA cycle at normal rates despite increased inhibitory phosphorylation (St Amand et al., 2000; Spriet and Heigenhauser, 2002). Our observations of enhanced glucose uptake, extracellular acidification and funneling of glucose carbons into lactate suggests that aerobic glycolysis is enhanced in cells lacking MCU. In addition, our results suggest that glucose-derived carbons are funneled into the TCA cycle through alternative pathways involving pyruvate carboxylation in MCU-KO cells. This pathway likely involves PC that generates m+3-labeled fumarate, malate, and aspartate from glucose.

Inhibition of transformed fibroblast proliferation *in vitro* by genetic deletion of MCU was associated with accumulation of the cells in S phase of the cell cycle. It was previously observed that MCU plays a role in mitotic progression (Cardenas et al., 2016) and cell-cycle progression from G1-S phase (Koval et al., 2019), both as a consequence of altered mitochondrial bioenergetics. Our results here suggest that stable deletion of MCU affects mitochondrial metabolism and the production of TCA cycle intermediates that fuel anabolic reactions critical for cell growth and proliferation (DeBerardinis et al., 2007; Mullen et al., 2011; Fendt et al., 2013; Birsoy et al., 2015; Lunt et al., 2015; Cardenas et al., 2016). Generation of biosynthetic substrates by mitochondria supports proliferation by supplementing aspartate for protein and nucleotide synthesis (Birsoy et al., 2015; Sullivan et al., 2015). The accumulation in S phase observed here is reminiscent of the proliferative phenotype caused by limited aspartate availability for *de novo* synthesis of pyrimidines (Gaglio et al., 2009; Birsoy et al., 2015; Lunt et al., 2015; Saqcena et al., 2015; Sullivan et al., 2015; Patel et al., 2016). Of note, we previously observed that an energetic crisis triggered by acute inhibition or silencing of InsP_3_R or MCU could be rescued by supplementation of exogenous nucleotides (Cardenas et al., 2010; Cardenas et al., 2016; Cardenas et al., 2020). Glutamine provides a nitrogen source for nucleotide synthesis, particularly important to prevent cell-cycle arrest in S phase (St Amand et al., 2000; Patel and Korotchkina, 2006; Saqcena et al., 2015). Thus, glutamine anaplerosis is often a limiting factor for cancer cell growth. Here, we found that deletion of MCU was associated with enhanced glutamine uptake and incorporation of its carbons into TCA-cycle intermediates. We observed a surprisingly high contribution of glutamine for the generation of aspartate pools. In addition, we observed a strong dependence of MCU-KO cell proliferation on glutamine availability. Enhanced lactate production, by fueling NAD^+^ production, may also contribute to enhanced aspartate synthesis by activating the cytosolic malate dehydrogenase to generate oxaloacetate that then drives aspartate synthesis by the aspartate aminotransferase GOT1 (Birsoy et al., 2015).

Despite altered metabolism observed as a consequence of MCU deletion, various bioenergetic parameters, including basal dehydrogenase activity, ROS production, ΔΨ_m_, and [Ca^2+^]_mit_, were independent of MCU in our studies. Lack of effect of MCU deletion on basal bioenergetics has been observed in some cell types (Pan et al., 2013; Luongo et al., 2015; Kwong et al., 2018), although suppression of mitochondrial Ca^2+^ uptake has been reported to disrupt ATP and ROS production and downregulate NAD^+^/NADH ratios in others (Tosatto et al., 2016; Ren et al., 2017; Young et al., 2022). Whereas respiration was unaffected, MCU deletion in our transformed fibroblasts was associated with elevated glycolysis and glutaminolysis, as previously observed in some mouse (Pan et al., 2013; Nichols et al., 2017; Gherardi et al., 2019) and cell (Young et al., 2022) models. An inverse correlation between mitochondrial Ca^2+^ uptake and glycolysis has been observed in epithelial carcinomas and ovarian cancer in which increased MICU1 expression promotes lactate accumulation (Chakraborty et al., 2017; Nemani et al., 2020). Several factors can promote a shift of cellular metabolism towards aerobic glycolysis (Gaude et al., 2018; Szibor et al., 2020; Luengo et al., 2021), including elevated AMPK activity (Young et al., 2022) that we and others previously found were associated with interruption of ER-to-mitochondria Ca^2+^ transfer (Cardenas et al., 2010; Luongo et al., 2015; Ren et al., 2017; Tomar et al., 2019; Zhao et al., 2019; Cardenas et al., 2020). Of note, forced reliance of HEK MCU-KO on the TCA cycle caused a reduction of ATP levels, activation of AMPK and a bioenergetic crisis that led to cell death (Young et al., 2022), similar to the responses of cancer cells to acute deletion of either MCU or InsP_3_R (Cardenas et al., 2016). Furthermore, chronic deletion of MCU in HEK cells results in cell death under conditions in which glycolysis is inhibited (Young et al., 2022). Enhanced glycolysis is broadly associated with proliferation (Diaz-Ruiz et al., 2011), suggesting that enhanced glycolysis in MCU-KO cells may be a compensatory mechanism to sustain cell growth. In previous studies, including our own, acute deletion or knockdown of MCU had significant effects on mitochondrial energy metabolism (Cardenas et al., 2016; Tosatto et al., 2016; Ren et al., 2017; Stejerean-Todoran et al., 2022). However, chronic deletion of MCU has been observed to not result in overt changes of basal mitochondrial metabolism (Pan et al., 2013; Kwong et al., 2015; Luongo et al., 2015; Hamilton et al., 2018; Koval et al., 2019; Wu et al., 2021). Discrepancies between chronic and acute MCU knockout/knockdown models is a recurring theme that might be explained by the activation of compensatory adaptations to maintain mitochondrial energy metabolism (Garbincius and Elrod, 2022). Whereas the apparent flux of glucose into the TCA cycle through PDH was largely unaltered by deletion of MCU, rescue of MCU enhanced this pathway, suggesting that re-wired metabolism in MCU-deleted cells enabled proliferation and survival by engagement of compensatory metabolic pathways.

In addition to identifying a critical role of MCU in tumor growth, we determined that MCU deletion in transformed fibroblasts diminished malignant capabilities *in vitro.* Our studies indicate that genetic deletion of MCU limits the ability of transformed fibroblasts to form clonally-derived spheres and invade, capabilities associated with initiation and progression of metastatic tumors (Uchida et al., 2010; Ishiguro et al., 2017), as previously observed (Tosatto et al., 2016). Previous studies have suggested a role for MCU as a promoter of invasion and metastasis in breast cancer and colorectal carcinoma-derived cell lines (Curry et al., 2013; Marchi et al., 2013; Tang et al., 2015; Cardenas et al., 2016; Tosatto et al., 2016; Ren et al., 2017; Yu et al., 2017; Liu et al., 2020). Thus, our results suggest that MCU supports cancer malignancy by promoting tumor growth as well as cell-biological functions involved in invasion and recurrence.

[Ca^2+^]_cyt_ signaling is a regulator of cell cycle progression (Humeau et al., 2018; Zhao and Pan, 2021). We observed a suppression of agonist-induced InsP_3_R-mediated [Ca^2+^]_cyt_ signals and sustained rise of [Ca^2+^]_cyt_ in MCU-KO fibroblasts. Our findings recapitulate those made by Koval et al., where genetic deletion of MCU in primary mouse fibroblasts decreased cell proliferation and delayed cell cycle progression through disruption of [Ca^2+^]_cyt_ transients (Koval et al., 2019). MCU helps sustain store-operated Ca^2+^ entry (SOCE) and [Ca^2+^]_cyt_ oscillations by rapid Ca^2+^ buffering, that are expected to be altered by absence of MCU (Kim and Usachev, 2009; Samanta et al., 2014; Zhao and Pan, 2021). [Ca^2+^]_cyt_ dynamics regulate essential processes that promote carcinogenesis and support malignancy. For example, dysregulated cytosolic Ca^2+^ signaling promotes the activation of Ca^2+^-sensitive proteins involved in upregulation of epithelial-to-mesenchymal transition, such as calmodulin (Ito et al., 1999; Norgard et al., 2021). Other studies implicate MCU-dependent clearance of cytoplasmic Ca^2+^ and Ca^2+^-sensitive CamKII activation in the regulation of cancer cell proliferation (Koval et al., 2019; Zhao and Pan, 2021), as well as inactivation and nuclear-translocation of the transcription factor NFAT (Kim and Usachev, 2009) implicated in cell survival, angiogenesis, and invasion (Qin et al., 2014). Accordingly, in addition to effects on metabolism, MCU deletion can also perturb cell-cycle progression through the dysregulation of [Ca^2+^]_cyt_ signaling. [Ca^2+^]_cyt_ regulates the activity of the aspartate/glutamate exchangers aralar and citrin, components of the malate-aspartate shuttle (MAS) (del Arco and Satrustegui, 1998; Del Arco et al., 2000; Palmieri et al., 2001; Contreras et al., 2007; Borst, 2020). Of particular importance, activation of the Aralar/MAS pathway promotes regeneration of cytosolic aspartate pools and its inhibition delays cell cycle progression (Contreras et al., 2007; Wang et al., 2016; Alkan and Bogner-Strauss, 2019; Infantino et al., 2019; Del Arco et al., 2023). As noted, cancer cells rely on mitochondrial aspartate for *de novo* pyrimidine synthesis to support uncontrolled proliferation (Brown et al., 2017; Wang et al., 2021), and our isotope labeling experiments revealed enhanced aspartate production. It is interesting to speculate that slow progression through S phase in MCU-KO cells might result from decreased MAS activity due to MCU-mediated alterations of [Ca^2+^]_cyt_ (Pardo et al., 2006; Patel et al., 2016; Wang et al., 2016; Diehl et al., 2022; Perez-Liebana et al., 2022; Del Arco et al., 2023).

In summary, we discovered that fibroblast cell transformation is associated with upregulation of MCU expression that results in altered mitochondrial Ca^2+^ uptake kinetics and suppression of inactivating-phosphorylation of PDH that was nevertheless not associated with enhanced pyruvate metabolism through PDH or OCR. Rather, transformation was associated with enhanced aerobic glycolysis and anaplerotic glutamine and glucose metabolism. Deletion of MCU strongly delayed tumor growth *in vivo* and decreased cell proliferation *in vitro* and *in vivo*. Mechanistically, this occurred by prolongation of the cell cycle, a strongly reduced capacity for sphere-forming ability and altered cytoplasmic Ca^2+^ signaling. Our results suggest that targeting MCU may have therapeutic implications in cancer.

## Data Availability

The original contributions presented in the study are included in the article/Supplementary Material, further inquiries can be directed to the corresponding author.

## References

[B1] Adusumilli R. Mallick P. (2017). Data conversion with ProteoWizard msConvert. Methods Mol. Biol. 1550, 339–368. 10.1007/978-1-4939-6747-6_23 28188540

[B2] Ahumada-Castro U. Puebla-Huerta A. Cuevas-Espinoza V. Lovy A. Cardenas J. C. (2021). Keeping zombies alive: The ER-mitochondria Ca^2+^ transfer in cellular senescence. Biochim. Biophys. Acta Mol. Cell Res. 1868 (11), 119099. 10.1016/j.bbamcr.2021.119099 34274397

[B3] Alkan H. F. Bogner-Strauss J. G. (2019). Maintaining cytosolic aspartate levels is a major function of the TCA cycle in proliferating cells. Mol. Cell Oncol. 6 (5), e1536843. 10.1080/23723556.2018.1536843 31528687 PMC6736317

[B4] Anwar S. Shamsi A. Mohammad T. Islam A. Hassan M. I. (2021). Targeting pyruvate dehydrogenase kinase signaling in the development of effective cancer therapy. Biochim. Biophys. Acta Rev. Cancer 1876 (1), 188568. 10.1016/j.bbcan.2021.188568 34023419

[B5] Asrani K. Murali S. Lam B. Na C. H. Phatak P. Sood A. (2019). mTORC1 feedback to AKT modulates lysosomal biogenesis through MiT/TFE regulation. J. Clin. Invest. 129 (12), 5584–5599. 10.1172/JCI128287 31527310 PMC6877313

[B6] Baughman J. M. Perocchi F. Girgis H. S. Plovanich M. Belcher-Timme C. A. Sancak Y. (2011). Integrative genomics identifies MCU as an essential component of the mitochondrial calcium uniporter. Nature 476 (7360), 341–345. 10.1038/nature10234 21685886 PMC3486726

[B7] Billings P. C. Sanzari J. K. Kennedy A. R. Cengel K. A. Seykora J. T. (2015). Comparative analysis of colorimetric staining in skin using open-source software. Exp. Dermatol 24 (2), 157–159. 10.1111/exd.12594 25393687 PMC4479192

[B8] Birsoy K. Wang T. Chen W. W. Freinkman E. Abu-Remaileh M. Sabatini D. M. (2015). An essential role of the mitochondrial electron transport chain in cell proliferation is to enable aspartate synthesis. Cell 162 (3), 540–551. 10.1016/j.cell.2015.07.016 26232224 PMC4522279

[B9] Borst P. (2020). The malate-aspartate shuttle (Borst cycle): How it started and developed into a major metabolic pathway. IUBMB Life 72 (11), 2241–2259. 10.1002/iub.2367 32916028 PMC7693074

[B10] Bowker-Kinley M. M. Davis W. I. Wu P. Harris R. A. Popov K. M. (1998). Evidence for existence of tissue-specific regulation of the mammalian pyruvate dehydrogenase complex. Biochem. J. 329 (1), 191–196. 10.1042/bj3290191 9405293 PMC1219031

[B11] Brown K. K. Spinelli J. B. Asara J. M. Toker A. (2017). Adaptive reprogramming of de novo pyrimidine synthesis is a metabolic vulnerability in triple-negative breast cancer. Cancer Discov. 7 (4), 391–399. 10.1158/2159-8290.CD-16-0611 28255083 PMC5380483

[B12] Bustos G. Ahumada-Castro U. Silva-Pavez E. Puebla A. Lovy A. Cesar Cardenas J. (2021). The ER-mitochondria Ca^2+^ signaling in cancer progression: Fueling the monster. Int. Rev. Cell Mol. Biol. 363, 49–121. 10.1016/bs.ircmb.2021.03.006 34392932

[B13] Cardenas C. Lovy A. Silva-Pavez E. Urra F. Mizzoni C. Ahumada-Castro U. (2020). Cancer cells with defective oxidative phosphorylation require endoplasmic reticulum-to-mitochondria Ca^2+^ transfer for survival. Sci. Signal 13 (640), eaay1212. 10.1126/scisignal.aay1212 32665411 PMC9387586

[B14] Cardenas C. Miller R. A. Smith I. Bui T. Molgo J. Muller M. (2010). Essential regulation of cell bioenergetics by constitutive InsP_3_ receptor Ca^2+^ transfer to mitochondria. Cell 142 (2), 270–283. 10.1016/j.cell.2010.06.007 20655468 PMC2911450

[B15] Cardenas C. Muller M. McNeal A. Lovy A. Jana F. Bustos G. (2016). Selective vulnerability of cancer cells by inhibition of Ca^2+^ transfer from endoplasmic reticulum to mitochondria. Cell Rep. 14 (10), 2313–2324. 10.1016/j.celrep.2016.02.030 26947070 PMC4794382

[B16] Chakraborty P. K. Mustafi S. B. Xiong X. Dwivedi S. K. D. Nesin V. Saha S. (2017). MICU1 drives glycolysis and chemoresistance in ovarian cancer. Nat. Commun. 8, 14634. 10.1038/ncomms14634 28530221 PMC5477507

[B17] Chambers M. C. Maclean B. Burke R. Amodei D. Ruderman D. L. Neumann S. (2012). A cross-platform toolkit for mass spectrometry and proteomics. Nat. Biotechnol. 30 (10), 918–920. 10.1038/nbt.2377 23051804 PMC3471674

[B18] Chaudhuri D. Sancak Y. Mootha V. K. Clapham D. E. (2013). MCU encodes the pore conducting mitochondrial calcium currents. Elife 2, e00704. 10.7554/eLife.00704 23755363 PMC3673318

[B19] Clasquin M. F. Melamud E. Rabinowitz J. D. (2012). LC-MS data processing with MAVEN: A metabolomic analysis and visualization engine. Curr. Protoc. Bioinforma. 14, 11. 10.1002/0471250953.bi1411s37 PMC4055029 22389014

[B20] Contreras L. Gomez-Puertas P. Iijima M. Kobayashi K. Saheki T. Satrustegui J. (2007). Ca^2+^ activation kinetics of the two aspartate-glutamate mitochondrial carriers, aralar and citrin: Role in the heart malate-aspartate NADH shuttle. J. Biol. Chem. 282 (10), 7098–7106. 10.1074/jbc.M610491200 17213189

[B21] Curry M. C. Peters A. A. Kenny P. A. Roberts-Thomson S. J. Monteith G. R. (2013). Mitochondrial calcium uniporter silencing potentiates caspase-independent cell death in MDA-MB-231 breast cancer cells. Biochem. Biophys. Res. Commun. 434 (3), 695–700. 10.1016/j.bbrc.2013.04.015 23602897

[B22] De Stefani D. Raffaello A. Teardo E. Szabo I. Rizzuto R. (2011). A forty-kilodalton protein of the inner membrane is the mitochondrial calcium uniporter. Nature 476 (7360), 336–340. 10.1038/nature10230 21685888 PMC4141877

[B23] DeBerardinis R. J. Lum J. J. Hatzivassiliou G. Thompson C. B. (2008a). The biology of cancer: Metabolic reprogramming fuels cell growth and proliferation. Cell Metab. 7 (1), 11–20. 10.1016/j.cmet.2007.10.002 18177721

[B24] DeBerardinis R. J. Mancuso A. Daikhin E. Nissim I. Yudkoff M. Wehrli S. (2007). Beyond aerobic glycolysis: Transformed cells can engage in glutamine metabolism that exceeds the requirement for protein and nucleotide synthesis. Proc. Natl. Acad. Sci. U. S. A. 104 (49), 19345–19350. 10.1073/pnas.0709747104 18032601 PMC2148292

[B25] Deberardinis R. J. Sayed N. Ditsworth D. Thompson C. B. (2008b). Brick by brick: Metabolism and tumor cell growth. Curr. Opin. Genet. Dev. 18 (1), 54–61. 10.1016/j.gde.2008.02.003 18387799 PMC2476215

[B26] Del Arco A. Agudo M. Satrustegui J. (2000). Characterization of a second member of the subfamily of calcium-binding mitochondrial carriers expressed in human non-excitable tissues. Biochem. J. 345 (3), 725–732. 10.1042/0264-6021:3450725 10642534 PMC1220810

[B27] Del Arco A. Gonzalez-Moreno L. Perez-Liebana I. Juaristi I. Gonzalez-Sanchez P. Contreras L. (2023). Regulation of neuronal energy metabolism by calcium: Role of MCU and Aralar/malate-aspartate shuttle. Biochim. Biophys. Acta Mol. Cell Res. 1870(5), 119468. 10.1016/j.bbamcr.2023.119468 36997074

[B28] del Arco A. Satrustegui J. (1998). Molecular cloning of Aralar, a new member of the mitochondrial carrier superfamily that binds calcium and is present in human muscle and brain. J. Biol. Chem. 273(36), 23327–23334. 10.1074/jbc.273.36.23327 9722566

[B29] Denton R. M. McCormack J. G. (1980). On the role of the calcium transport cycle in heart and other mammalian mitochondria. FEBS Lett. 119 (1), 1–8. 10.1016/0014-5793(80)80986-0 7000543

[B30] Denton R. M. (2009). Regulation of mitochondrial dehydrogenases by calcium ions. Biochim. Biophys. Acta 1787 (11), 1309–1316. 10.1016/j.bbabio.2009.01.005 19413950

[B31] Diaz-Ruiz R. Rigoulet M. Devin A. (2011). The Warburg and Crabtree effects: On the origin of cancer cell energy metabolism and of yeast glucose repression. Biochim. Biophys. Acta 1807 (6), 568–576. 10.1016/j.bbabio.2010.08.010 20804724

[B32] Diehl F. F. Miettinen T. P. Elbashir R. Nabel C. S. Darnell A. M. Do B. T. (2022). Nucleotide imbalance decouples cell growth from cell proliferation. Nat. Cell Biol. 24 (8), 1252–1264. 10.1038/s41556-022-00965-1 35927450 PMC9359916

[B33] Fendt S. M. Bell E. L. Keibler M. A. Olenchock B. A. Mayers J. R. Wasylenko T. M. (2013). Reductive glutamine metabolism is a function of the alpha-ketoglutarate to citrate ratio in cells. Nat. Commun. 4, 2236. 10.1038/ncomms3236 23900562 PMC3934748

[B34] Foskett J. K. (2010). Inositol trisphosphate receptor Ca^2+^ release channels in neurological diseases. Pflugers Arch. 460 (2), 481–494. 10.1007/s00424-010-0826-0 20383523 PMC2893360

[B35] Gaglio D. Soldati C. Vanoni M. Alberghina L. Chiaradonna F. (2009). Glutamine deprivation induces abortive s-phase rescued by deoxyribonucleotides in k-ras transformed fibroblasts. PLoS One 4 (3), e4715. 10.1371/journal.pone.0004715 19262748 PMC2650790

[B36] Ganapathy-Kanniappan S. Geschwind J. F. (2013). Tumor glycolysis as a target for cancer therapy: Progress and prospects. Mol. Cancer 12, 152. 10.1186/1476-4598-12-152 24298908 PMC4223729

[B37] Garbincius J. F. Elrod J. W. (2022). Mitochondrial calcium exchange in physiology and disease. Physiol. Rev. 102 (2), 893–992. 10.1152/physrev.00041.2020 34698550 PMC8816638

[B38] Garbincius J. F. Luongo T. S. Elrod J. W. (2020). The debate continues - what is the role of MCU and mitochondrial calcium uptake in the heart? J. Mol. Cell Cardiol. 143, 163–174. 10.1016/j.yjmcc.2020.04.029 32353353 PMC7938348

[B39] Gaude E. Schmidt C. Gammage P. A. Dugourd A. Blacker T. Chew S. P. (2018). NADH shuttling couples cytosolic reductive carboxylation of glutamine with glycolysis in cells with mitochondrial dysfunction. Mol. Cell 69 (4), 581–593. 10.1016/j.molcel.2018.01.034 29452638 PMC5823973

[B40] Gherardi G. Nogara L. Ciciliot S. Fadini G. P. Blaauw B. Braghetta P. (2019). Loss of mitochondrial calcium uniporter rewires skeletal muscle metabolism and substrate preference. Cell Death Differ. 26 (2), 362–381. 10.1038/s41418-018-0191-7 30232375 PMC6329801

[B41] Guerra M. T. Florentino R. M. Franca A. Lima Filho A. C. Dos Santos M. L. Fonseca R. C. (2019). Expression of the type 3 InsP_3_ receptor is a final common event in the development of hepatocellular carcinoma. Gut 68 (9), 1676–1687. 10.1136/gutjnl-2018-317811 31315892 PMC7087395

[B42] Gui D. Y. Sullivan L. B. Luengo A. Hosios A. M. Bush L. N. Gitego N. (2016). Environment dictates dependence on mitochondrial complex I for NAD^+^ and aspartate production and determines cancer cell sensitivity to metformin. Cell Metab. 24 (5), 716–727. 10.1016/j.cmet.2016.09.006 27746050 PMC5102768

[B43] Hall D. D. Wu Y. Domann F. E. Spitz D. R. Anderson M. E. (2014). Mitochondrial calcium uniporter activity is dispensable for MDA-MB-231 breast carcinoma cell survival. PLoS One 9 (5), e96866. 10.1371/journal.pone.0096866 24802861 PMC4011874

[B44] Hamilton S. Terentyeva R. Kim T. Y. Bronk P. Clements R. T. J O. U. (2018). Pharmacological modulation of mitochondrial Ca^2+^ content regulates sarcoplasmic reticulum Ca^2+^ release via oxidation of the ryanodine receptor by mitochondria-derived reactive oxygen species. Front. Physiol. 9, 1831. 10.3389/fphys.2018.01831 30622478 PMC6308295

[B45] Hanahan D. Weinberg R. A. (2011). Hallmarks of cancer: The next generation. Cell 144 (5), 646–674. 10.1016/j.cell.2011.02.013 21376230

[B46] Harrington J. L. Murphy E. (2015). The mitochondrial calcium uniporter: Mice can live and die without it. J. Mol. Cell Cardiol. 78, 46–53. 10.1016/j.yjmcc.2014.10.013 25451167 PMC4305342

[B47] Hatzivassiliou G. Zhao F. Bauer D. E. Andreadis C. Shaw A. N. Dhanak D. (2005). ATP citrate lyase inhibition can suppress tumor cell growth. Cancer Cell 8 (4), 311–321. 10.1016/j.ccr.2005.09.008 16226706

[B48] Herst P. M. Carson G. M. Eccles D. A. Berridge M. V. (2022). Bioenergetic and metabolic adaptation in tumor progression and metastasis. Front. Oncol. 12, 857686. 10.3389/fonc.2022.857686 35372069 PMC8968714

[B49] Hoang G. Zhang C. Attarwala N. Jung J. G. Cooper A. J. L. Le A. (2021). Uncovering metabolic reservoir cycles in MYC-transformed lymphoma B cells using stable isotope resolved metabolomics. Anal. Biochem. 632, 114206. 10.1016/j.ab.2021.114206 33894159

[B50] Huang M. S. Adebanjo O. A. Awumey E. Biswas G. Koval A. Sodam B. R. (2000). IP(3), IP(3) receptor, and cellular senescence. Am. J. Physiol. Ren. Physiol. 278 (4), F576–F584. 10.1152/ajprenal.2000.278.4.F576 10751218

[B51] Humeau J. Bravo-San Pedro J. M. Vitale I. Nunez L. Villalobos C. Kroemer G. (2018). Calcium signaling and cell cycle: Progression or death. Cell Calcium 70, 3–15. 10.1016/j.ceca.2017.07.006 28801101

[B52] Infantino V. Dituri F. Convertini P. Santarsiero A. Palmieri F. Todisco S. (2019). Epigenetic upregulation and functional role of the mitochondrial aspartate/glutamate carrier isoform 1 in hepatocellular carcinoma. Biochim. Biophys. Acta Mol. Basis Dis. 1865 (1), 38–47. 10.1016/j.bbadis.2018.10.018 30321589

[B53] Ishiguro T. Ohata H. Sato A. Yamawaki K. Enomoto T. Okamoto K. (2017). Tumor-derived spheroids: Relevance to cancer stem cells and clinical applications. Cancer Sci. 108 (3), 283–289. 10.1111/cas.13155 28064442 PMC5378268

[B54] Ito K. Okamoto I. Araki N. Kawano Y. Nakao M. Fujiyama S. (1999). Calcium influx triggers the sequential proteolysis of extracellular and cytoplasmic domains of E-cadherin, leading to loss of beta-catenin from cell-cell contacts. Oncogene 18 (50), 7080–7090. 10.1038/sj.onc.1203191 10597309

[B55] Johnson S. Chen H. Lo P. K. (2013). *In vitro* tumorsphere formation assays. Bio Protoc. 3 (3), e325. 10.21769/bioprotoc.325 PMC4972326 27500184

[B56] Jose C. Bellance N. Rossignol R. (2011). Choosing between glycolysis and oxidative phosphorylation: A tumor's dilemma? Biochim. Biophys. Acta 1807 (6), 552–561. 10.1016/j.bbabio.2010.10.012 20955683

[B57] Keyomarsi K. Sandoval L. Band V. Pardee A. B. (1991). Synchronization of tumor and normal cells from G1 to multiple cell cycles by lovastatin. Cancer Res. 51 (13), 3602–3609. 1711413

[B58] Kim M. S. Usachev Y. M. (2009). Mitochondrial Ca^2+^ cycling facilitates activation of the transcription factor NFAT in sensory neurons. J. Neurosci. 29 (39), 12101–12114. 10.1523/JNEUROSCI.3384-09.2009 19793968 PMC2805078

[B59] Koval O. M. Nguyen E. K. Santhana V. Fidler T. P. Sebag S. C. Rasmussen T. P. (2019). Loss of MCU prevents mitochondrial fusion in G1-S phase and blocks cell cycle progression and proliferation. Sci. Signal 12 (579), eaav1439. 10.1126/scisignal.aav1439 31040260 PMC6768401

[B60] Kwong J. Q. Huo J. Bround M. J. Boyer J. G. Schwanekamp J. A. Ghazal N. (2018). The mitochondrial calcium uniporter underlies metabolic fuel preference in skeletal muscle. JCI Insight 3 (22), e121689. 10.1172/jci.insight.121689 30429366 PMC6302934

[B61] Kwong J. Q. Lu X. Correll R. N. Schwanekamp J. A. Vagnozzi R. J. Sargent M. A. (2015). The mitochondrial calcium uniporter selectively matches metabolic output to acute contractile stress in the heart. Cell Rep. 12 (1), 15–22. 10.1016/j.celrep.2015.06.002 26119742 PMC4497842

[B62] Li X. Spelat R. Bartolini A. Cesselli D. Ius T. Skrap M. (2020). Mechanisms of malignancy in glioblastoma cells are linked to mitochondrial Ca^2+^ uniporter upregulation and higher intracellular Ca^2+^ levels. J. Cell Sci. 133 (6), jcs237503. 10.1242/jcs.237503 32051286

[B63] Liberti M. V. Locasale J. W. (2016). The warburg effect: How does it benefit cancer cells? Trends Biochem. Sci. 41 (3), 211–218. 10.1016/j.tibs.2015.12.001 26778478 PMC4783224

[B64] Liu Y. Jin M. Wang Y. Zhu J. Tan R. Zhao J. (2020). MCU-induced mitochondrial calcium uptake promotes mitochondrial biogenesis and colorectal cancer growth. Signal Transduct. Target Ther. 5 (1), 59. 10.1038/s41392-020-0155-5 32371956 PMC7200750

[B65] Luengo A. Li Z. Gui D. Y. Sullivan L. B. Zagorulya M. Do B. T. (2021). Increased demand for NAD^+^ relative to ATP drives aerobic glycolysis. Mol. Cell 81 (4), 691–707.e6. 10.1016/j.molcel.2020.12.012 33382985 PMC8315838

[B66] Lunt S. Y. Muralidhar V. Hosios A. M. Israelsen W. J. Gui D. Y. Newhouse L. (2015). Pyruvate kinase isoform expression alters nucleotide synthesis to impact cell proliferation. Mol. Cell 57 (1), 95–107. 10.1016/j.molcel.2014.10.027 25482511 PMC4289430

[B67] Lunt S. Y. Vander Heiden M. G. (2011). Aerobic glycolysis: Meeting the metabolic requirements of cell proliferation. Annu. Rev. Cell Dev. Biol. 27, 441–464. 10.1146/annurev-cellbio-092910-154237 21985671

[B68] Luongo T. S. Lambert J. P. Yuan A. Zhang X. Gross P. Song J. (2015). The mitochondrial calcium uniporter matches energetic supply with cardiac workload during stress and modulates permeability transition. Cell Rep. 12 (1), 23–34. 10.1016/j.celrep.2015.06.017 26119731 PMC4517182

[B69] Mallilankaraman K. Doonan P. Cardenas C. Chandramoorthy H. C. Muller M. Miller R. (2012). MICU1 is an essential gatekeeper for MCU-mediated mitochondrial Ca^2+^ uptake that regulates cell survival. Cell 151 (3), 630–644. 10.1016/j.cell.2012.10.011 23101630 PMC3486697

[B70] Marchi S. Corricelli M. Branchini A. Vitto V. A. M. Missiroli S. Morciano G. (2019). Akt-mediated phosphorylation of MICU1 regulates mitochondrial Ca^2+^ levels and tumor growth. EMBO J. 38 (2), e99435. 10.15252/embj.201899435 30504268 PMC6331721

[B71] Marchi S. Lupini L. Patergnani S. Rimessi A. Missiroli S. Bonora M. (2013). Downregulation of the mitochondrial calcium uniporter by cancer-related miR-25. Curr. Biol. 23 (1), 58–63. 10.1016/j.cub.2012.11.026 23246404 PMC3540261

[B72] Melamud E. Vastag L. Rabinowitz J. D. (2010). Metabolomic analysis and visualization engine for LC-MS data. Anal. Chem. 82 (23), 9818–9826. 10.1021/ac1021166 21049934 PMC5748896

[B73] Miao Y. Wang X. Lai Y. Lin W. Huang Y. Yin H. (2021). Mitochondrial calcium uniporter promotes cell proliferation and migration in esophageal cancer. Oncol. Lett. 22 (3), 686. 10.3892/ol.2021.12947 34434285 PMC8335723

[B74] Mound A. Vautrin-Glabik A. Foulon A. Botia B. Hague F. Parys J. B. (2017). Downregulation of type 3 inositol (1,4,5)-trisphosphate receptor decreases breast cancer cell migration through an oscillatory Ca^2+^ signal. Oncotarget 8 (42), 72324–72341. 10.18632/oncotarget.20327 29069790 PMC5641133

[B75] Mullen A. R. Wheaton W. W. Jin E. S. Chen P. H. Sullivan L. B. Cheng T. (2011). Reductive carboxylation supports growth in tumour cells with defective mitochondria. Nature 481 (7381), 385–388. 10.1038/nature10642 22101431 PMC3262117

[B76] Nemani N. Dong Z. Daw C. C. Madaris T. R. Ramachandran K. Enslow B. T. (2020). Mitochondrial pyruvate and fatty acid flux modulate MICU1-dependent control of MCU activity. Sci. Signal 13 (628), eaaz6206. 10.1126/scisignal.aaz6206 32317369 PMC7667998

[B77] Nichols M. Elustondo P. A. Warford J. Thirumaran A. Pavlov E. V. Robertson G. S. (2017). Global ablation of the mitochondrial calcium uniporter increases glycolysis in cortical neurons subjected to energetic stressors. J. Cereb. Blood Flow. Metab. 37 (8), 3027–3041. 10.1177/0271678X16682250 27909264 PMC5536808

[B78] Norgard R. J. Pitarresi J. R. Maddipati R. Aiello-Couzo N. M. Balli D. Li J. (2021). Calcium signaling induces a partial EMT. EMBO Rep. 22 (9), e51872. 10.15252/embr.202051872 34324787 PMC8419705

[B79] Paliwal S. Fiumera H. L. Mohanty S. (2021). Stem cell plasticity and regenerative potential regulation through Ca^2+^-mediated mitochondrial nuclear crosstalk. Mitochondrion 56, 1–14. 10.1016/j.mito.2020.10.002 33059088

[B80] Palmieri L. Pardo B. Lasorsa F. M. del Arco A. Kobayashi K. Iijima M. (2001). Citrin and aralar1 are Ca^2+^-stimulated aspartate/glutamate transporters in mitochondria. EMBO J. 20 (18), 5060–5069. 10.1093/emboj/20.18.5060 11566871 PMC125626

[B81] Pan X. Liu J. Nguyen T. Liu C. Sun J. Teng Y. (2013). The physiological role of mitochondrial calcium revealed by mice lacking the mitochondrial calcium uniporter. Nat. Cell Biol. 15 (12), 1464–1472. 10.1038/ncb2868 24212091 PMC3852190

[B82] Pardo B. Contreras L. Serrano A. Ramos M. Kobayashi K. Iijima M. (2006). Essential role of aralar in the transduction of small Ca^2+^ signals to neuronal mitochondria. J. Biol. Chem. 281 (2), 1039–1047. 10.1074/jbc.M507270200 16269409

[B83] Patel D. Menon D. Bernfeld E. Mroz V. Kalan S. Loayza D. (2016). Aspartate rescues S-phase arrest caused by suppression of glutamine utilization in KRas-driven cancer cells. J. Biol. Chem. 291 (17), 9322–9329. 10.1074/jbc.M115.710145 26921316 PMC4861495

[B84] Patel M. S. Korotchkina L. G. (2006). Regulation of the pyruvate dehydrogenase complex. Biochem. Soc. Trans. 34 (2), 217–222. 10.1042/BST20060217 16545080

[B85] Perez-Liebana I. Juaristi I. Gonzalez-Sanchez P. Gonzalez-Moreno L. Rial E. Podunavac M. (2022). A Ca^2+^-dependent mechanism boosting glycolysis and OXPHOS by activating aralar-malate-aspartate shuttle, upon neuronal stimulation. J. Neurosci. 42 (19), 3879–3895. 10.1523/JNEUROSCI.1463-21.2022 35387872 PMC9097769

[B86] Qin J. J. Nag S. Wang W. Zhou J. Zhang W. D. Wang H. (2014). NFAT as cancer target: Mission possible? Biochim. Biophys. Acta 1846 (2), 297–311. 10.1016/j.bbcan.2014.07.009 25072963 PMC4710172

[B87] Reitzer L. J. Wice B. M. Kennell D. (1979). Evidence that glutamine, not sugar, is the major energy source for cultured HeLa cells. J. Biol. Chem. 254 (8), 2669–2676. 10.1016/s0021-9258(17)30124-2 429309

[B88] Ren T. Zhang H. Wang J. Zhu J. Jin M. Wu Y. (2017). MCU-dependent mitochondrial Ca^2+^ inhibits NAD^+^/SIRT3/SOD2 pathway to promote ROS production and metastasis of HCC cells. Oncogene 36 (42), 5897–5909. 10.1038/onc.2017.167 28650465

[B89] Resende R. R. Adhikari A. da Costa J. L. Lorencon E. Ladeira M. S. Guatimosim S. (2010). Influence of spontaneous calcium events on cell-cycle progression in embryonal carcinoma and adult stem cells. Biochim. Biophys. Acta 1803 (2), 246–260. 10.1016/j.bbamcr.2009.11.008 19958796

[B90] Rezuchova I. Hudecova S. Soltysova A. Matuskova M. Durinikova E. Chovancova B. (2019). Type 3 inositol 1,4,5-trisphosphate receptor has antiapoptotic and proliferative role in cancer cells. Cell Death Dis. 10 (3), 186. 10.1038/s41419-019-1433-4 30796197 PMC6385365

[B91] Rizzuto R. Pinton P. Carrington W. Fay F. S. Fogarty K. E. Lifshitz L. M. (1998). Close contacts with the endoplasmic reticulum as determinants of mitochondrial Ca^2+^ responses. Science 280 (5370), 1763–1766. 10.1126/science.280.5370.1763 9624056

[B92] Roche T. E. Hiromasa Y. Turkan A. Gong X. Peng T. Yan X. (2003). Essential roles of lipoyl domains in the activated function and control of pyruvate dehydrogenase kinases and phosphatase isoform 1. Eur. J. Biochem. 270 (6), 1050–1056. 10.1046/j.1432-1033.2003.03468.x 12631265

[B93] Samanta K. Douglas S. Parekh A. B. (2014). Mitochondrial calcium uniporter MCU supports cytoplasmic Ca^2+^ oscillations, store-operated Ca^2+^ entry and Ca^2+^-dependent gene expression in response to receptor stimulation. PLoS One 9 (7), e101188. 10.1371/journal.pone.0101188 25004162 PMC4086884

[B94] Saqcena M. Mukhopadhyay S. Hosny C. Alhamed A. Chatterjee A. Foster D. A. (2015). Blocking anaplerotic entry of glutamine into the TCA cycle sensitizes K-Ras mutant cancer cells to cytotoxic drugs. Oncogene 34 (20), 2672–2680. 10.1038/onc.2014.207 25023699 PMC4418945

[B95] Sasaki R. Narisawa-Saito M. Yugawa T. Fujita M. Tashiro H. Katabuchi H. (2009). Oncogenic transformation of human ovarian surface epithelial cells with defined cellular oncogenes. Carcinogenesis 30 (3), 423–431. 10.1093/carcin/bgp007 19126650

[B124] Schindelin J. Arganda-Carreras I. Frise E. Kaynig V. Longair M. Pietzsch T. (2012). Fiji: an open-source platform for biological-image analysis. Nat. Methods 9, 676–682. 10.1038/nmeth.2019 22743772 PMC3855844

[B96] Shi J. L. Fu L. Wang W. D. (2015). High expression of inositol 1,4,5-trisphosphate receptor, type 2 (ITPR2) as a novel biomarker for worse prognosis in cytogenetically normal acute myeloid leukemia. Oncotarget 6 (7), 5299–5309. 10.18632/oncotarget.3024 25779662 PMC4467150

[B97] Shibao K. Fiedler M. J. Nagata J. Minagawa N. Hirata K. Nakayama Y. (2010). The type III inositol 1,4,5-trisphosphate receptor is associated with aggressiveness of colorectal carcinoma. Cell Calcium 48 (6), 315–323. 10.1016/j.ceca.2010.09.005 21075448 PMC3572849

[B98] Spriet L. L. Heigenhauser G. J. (2002). Regulation of pyruvate dehydrogenase (PDH) activity in human skeletal muscle during exercise. Exerc Sport Sci. Rev. 30 (2), 91–95. 10.1097/00003677-200204000-00009 11991544

[B99] St Amand T. A. Spriet L. L. Jones N. L. Heigenhauser G. J. (2000). Pyruvate overrides inhibition of PDH during exercise after a low-carbohydrate diet. Am. J. Physiol. Endocrinol. Metab. 279 (2), E275–E283. 10.1152/ajpendo.2000.279.2.E275 10913026

[B100] Stejerean-Todoran I. Zimmermann K. Gibhardt C. S. Vultur A. Ickes C. Shannan B. (2022). MCU controls melanoma progression through a redox-controlled phenotype switch. EMBO Rep. 23 (11), e54746. 10.15252/embr.202254746 36156348 PMC9638851

[B101] Su X. Lu W. Rabinowitz J. D. (2017). Metabolite spectral accuracy on orbitraps. Anal. Chem. 89 (11), 5940–5948. 10.1021/acs.analchem.7b00396 28471646 PMC5748891

[B102] Sullivan L. B. Gui D. Y. Hosios A. M. Bush L. N. Freinkman E. Vander Heiden M. G. (2015). Supporting aspartate biosynthesis is an essential function of respiration in proliferating cells. Cell 162 (3), 552–563. 10.1016/j.cell.2015.07.017 26232225 PMC4522278

[B103] Sullivan M. R. Vander Heiden M. G. (2019). Determinants of nutrient limitation in cancer. Crit. Rev. Biochem. Mol. Biol. 54 (3), 193–207. 10.1080/10409238.2019.1611733 31162937 PMC6715536

[B104] Suzuki J. Kanemaru K. Ishii K. Ohkura M. Okubo Y. Iino M. (2014). Imaging intraorganellar Ca^2+^ at subcellular resolution using CEPIA. Nat. Commun. 5, 4153. 10.1038/ncomms5153 24923787 PMC4082642

[B105] Szibor M. Gizatullina Z. Gainutdinov T. Endres T. Debska-Vielhaber G. Kunz M. (2020). Cytosolic, but not matrix, calcium is essential for adjustment of mitochondrial pyruvate supply. J. Biol. Chem. 295 (14), 4383–4397. 10.1074/jbc.RA119.011902 32094224 PMC7135991

[B106] Tang S. Wang X. Shen Q. Yang X. Yu C. Cai C. (2015). Mitochondrial Ca^2+^ uniporter is critical for store-operated Ca^2+^ entry-dependent breast cancer cell migration. Biochem. Biophys. Res. Commun. 458 (1), 186–193. 10.1016/j.bbrc.2015.01.092 25640838

[B107] Tomar D. Jana F. Dong Z. Quinn W. J. Jadiya P. Breves S. L. (2019). Blockade of MCU-mediated Ca^2+^ uptake perturbs lipid metabolism via PP4-dependent AMPK dephosphorylation. Cell Rep. 26 (13), 3709–3725. 10.1016/j.celrep.2019.02.107 30917323 PMC6512325

[B108] Tosatto A. Sommaggio R. Kummerow C. Bentham R. B. Blacker T. S. Berecz T. (2016). The mitochondrial calcium uniporter regulates breast cancer progression via HIF-1α. EMBO Mol. Med. 8 (5), 569–585. 10.15252/emmm.201606255 27138568 PMC4864890

[B109] Uchida Y. Tanaka S. Aihara A. Adikrisna R. Yoshitake K. Matsumura S. (2010). Analogy between sphere forming ability and stemness of human hepatoma cells. Oncol. Rep. 24 (5), 1147–1151. 10.3892/or_00000966 20878104

[B110] Umemoto T. Hashimoto M. Matsumura T. Nakamura-Ishizu A. Suda T. (2018). Ca^2+^-mitochondria axis drives cell division in hematopoietic stem cells. J. Exp. Med. 215 (8), 2097–2113. 10.1084/jem.20180421 29946000 PMC6080917

[B111] Vanhove K. Graulus G. J. Mesotten L. Thomeer M. Derveaux E. Noben J. P. (2019). The metabolic landscape of lung cancer: New insights in a disturbed glucose metabolism. Front. Oncol. 9, 1215. 10.3389/fonc.2019.01215 31803611 PMC6873590

[B112] Wang C. Chen H. Zhang M. Zhang J. Wei X. Ying W. (2016). Malate-aspartate shuttle inhibitor aminooxyacetic acid leads to decreased intracellular ATP levels and altered cell cycle of C6 glioma cells by inhibiting glycolysis. Cancer Lett. 378 (1), 1–7. 10.1016/j.canlet.2016.05.001 27157912

[B113] Wang W. Cui J. Ma H. Lu W. Huang J. (2021). Targeting pyrimidine metabolism in the era of precision cancer medicine. Front. Oncol. 11, 684961. 10.3389/fonc.2021.684961 34123854 PMC8194085

[B114] Wang X. Song X. Cheng G. Zhang J. Dong L. Bai J. (2020). The regulatory mechanism and biological significance of mitochondrial calcium uniporter in the migration, invasion, angiogenesis and growth of gastric cancer. Onco Targets Ther. 13, 11781–11794. 10.2147/OTT.S262049 33235465 PMC7680189

[B115] Weissenrieder J. S. Reed J. L. Green M. V. Moldovan G. L. Koubek E. J. Neighbors J. D. (2020). The dopamine D2 receptor contributes to the spheroid formation behavior of U87 glioblastoma cells. Pharmacology 105 (1-2), 19–27. 10.1159/000502562 31645049 PMC10777736

[B116] Wise D. R. DeBerardinis R. J. Mancuso A. Sayed N. Zhang X. Y. Pfeiffer H. K.(2008). Myc regulates a transcriptional program that stimulates mitochondrial glutaminolysis and leads to glutamine addiction. Proc. Natl. Acad. Sci. U. S. A. 105 (48), 18782–18787. 10.1073/pnas.0810199105 19033189 PMC2596212

[B117] Wu R. Zuo W. Xu X. Bi L. Zhang C. Chen H.(2021). MCU that is transcriptionally regulated by Nrf2 augments malignant biological behaviors in oral squamous cell carcinoma cells. Biomed. Res. Int. 2021, 6650791. 10.1155/2021/6650791 34189138 PMC8195654

[B118] Yao C. H. Wang R. Wang Y. Kung C. P. Weber J. D. Patti G. J. (2019). Mitochondrial fusion supports increased oxidative phosphorylation during cell proliferation. Elife 8, e41351. 10.7554/eLife.41351 30694178 PMC6351101

[B119] Young M. P. Schug Z. T. Booth D. M. Yule D. I. Mikoshiba K. Hajnomicronczky G. (2022). Metabolic adaptation to the chronic loss of Ca^2+^ signaling induced by KO of IP3 receptors or the mitochondrial Ca^2+^ uniporter. J. Biol. Chem. 298 (1), 101436. 10.1016/j.jbc.2021.101436 34801549 PMC8672050

[B120] Yu C. Wang Y. Peng J. Shen Q. Chen M. Tang W. (2017). Mitochondrial calcium uniporter as a target of microRNA-340 and promoter of metastasis via enhancing the Warburg effect. Oncotarget 8 (48), 83831–83844. 10.18632/oncotarget.19747 29137386 PMC5663558

[B121] Zhao H. Li T. Wang K. Zhao F. Chen J. Xu G. (2019). AMPK-mediated activation of MCU stimulates mitochondrial Ca^2+^ entry to promote mitotic progression. Nat. Cell Biol. 21 (4), 476–486. 10.1038/s41556-019-0296-3 30858581

[B122] Zhao H. Pan X. (2021). Mitochondrial Ca^2+^ and cell cycle regulation. Int. Rev. Cell Mol. Biol. 362, 171–207. 10.1016/bs.ircmb.2021.02.015 34253295

[B123] Zhao Y. Wang Y. Zhao J. Zhang Z. Jin M. Zhou F. (2021). PDE2 inhibits PKA-mediated phosphorylation of TFAM to promote mitochondrial Ca^2+^-induced colorectal cancer growth. Front. Oncol. 11, 663778. 10.3389/fonc.2021.663778 34235078 PMC8256694

