# Supplementary material for: The mitochondrial Ca2+ channel MCU is critical for tumor growth by supporting cell cycle progression and proliferation
Source: Front Cell Dev Biol. 2023 Jun 8;11:1082213. doi: 10.3389/fcell.2023.1082213 (PMC10285664; doi:10.3389/fcell.2023.1082213)
Supplement: Supplementary file 1 [file DataSheet1.docx]

Supplementary Material

# Supplementary Figures

**Supplementary Figure 1.** Transformation of fibroblasts increases mitochondrial mass, upregulates MCU expression and enhances mitochondrial Ca^2+^ uptake. (**A**) Representative immunoblots of HSP60, Tim23 and tubulin in immortalized and transformed fibroblasts. (**B**) HSP60 levels normalized to tubulin detected on the same blots. Values expressed as fluorescence units (f.a.u*1000) (mean ± SEM, n = 3, ***p < 0.001, Student’s t-test). (**C**) Tim23 levels normalized to tubulin detected on the same blots. Values expressed as fluorescence units (f.a.u*1000) (mean ± SEM, n = 3, *p < 0.05, Student’s t-test). (**D**) Representative immunoblots of MCU and tubulin in WT- and transformed-fibroblasts. (**E**) MCU levels normalized to tubulin on same blots. Values expressed as fluorescence units (f.a.u*1000) (mean ± SEM, n = 3, ns = non-significant, Student’s t-test). (**F**) Average traces of [Ca^2+^]_cyt_ in suspensions of permeabilized WT- and transformed-fibroblasts (mean ± SEM). (**G**) Mitochondrial Ca^2+^ uptake rates of permeabilized WT- and transformed-fibroblasts (mean ± SEM, n = 4, **p < 0.01, Student’s t-test). (**H**) ATP-stimulated [Ca^2+^]_m_ measured with the genetically-encoded indicator CEPIA3mt. Fluorescence (F) normalized to averaged values between T = 0 and 1 min (F_0_) to obtain reported F/F_0_ (n = number of cells; mean ± SEM). (**I**) Basal O_2_ consumption rates (OCR) of transformed and immortalized fibroblasts (mean ± SEM, n = 8, ns = non-significant, Student’s t-test). (**J**) Maximal uncoupled OCR of transformed and immortalized fibroblasts (mean ± SEM, n = 4, ns = non-significant, Student’s t-test). (**K**) Maximal uncoupled OCR of transformed and immortalized fibroblasts after acute stimulation of OCR by ATP (mean ± SEM, n = 4, ns = non-significant, Student’s t-test). (**L**) Representative immunoblots of MCU and tubulin in HEK293T WT, MCU-KO, and MCU-rescue cells. (**M**) MCU levels normalized to tubulin expression detected on same blots (mean ± SEM, n = 3, ***p < 0.001, one-way ANOVA). (**N**) Average traces (n = 3) of [Ca^2+^]_cyt_ in suspensions of permeabilized HEK293T WT, HEK293T MCU-KO, and HEK293T MCU-rescue cells (mean ± SEM). (**O**) Mitochondrial Ca^2+^ uptake rates of permeabilized HEK293T WT, MCU-KO, and MCU-rescue cells (mean ± SEM, n = 3, **p < 0.01, ***p < 0.001, one-way ANOVA). (**P**) Normalized ΔΨ_m_ (f.a.u) in permeabilized suspensions of HEK293T WT (n = 4), MCU-KO (n = 4), and MCU-rescue (n = 3) cells (mean ± SEM, ns = non-significant, one-way ANOVA).

##


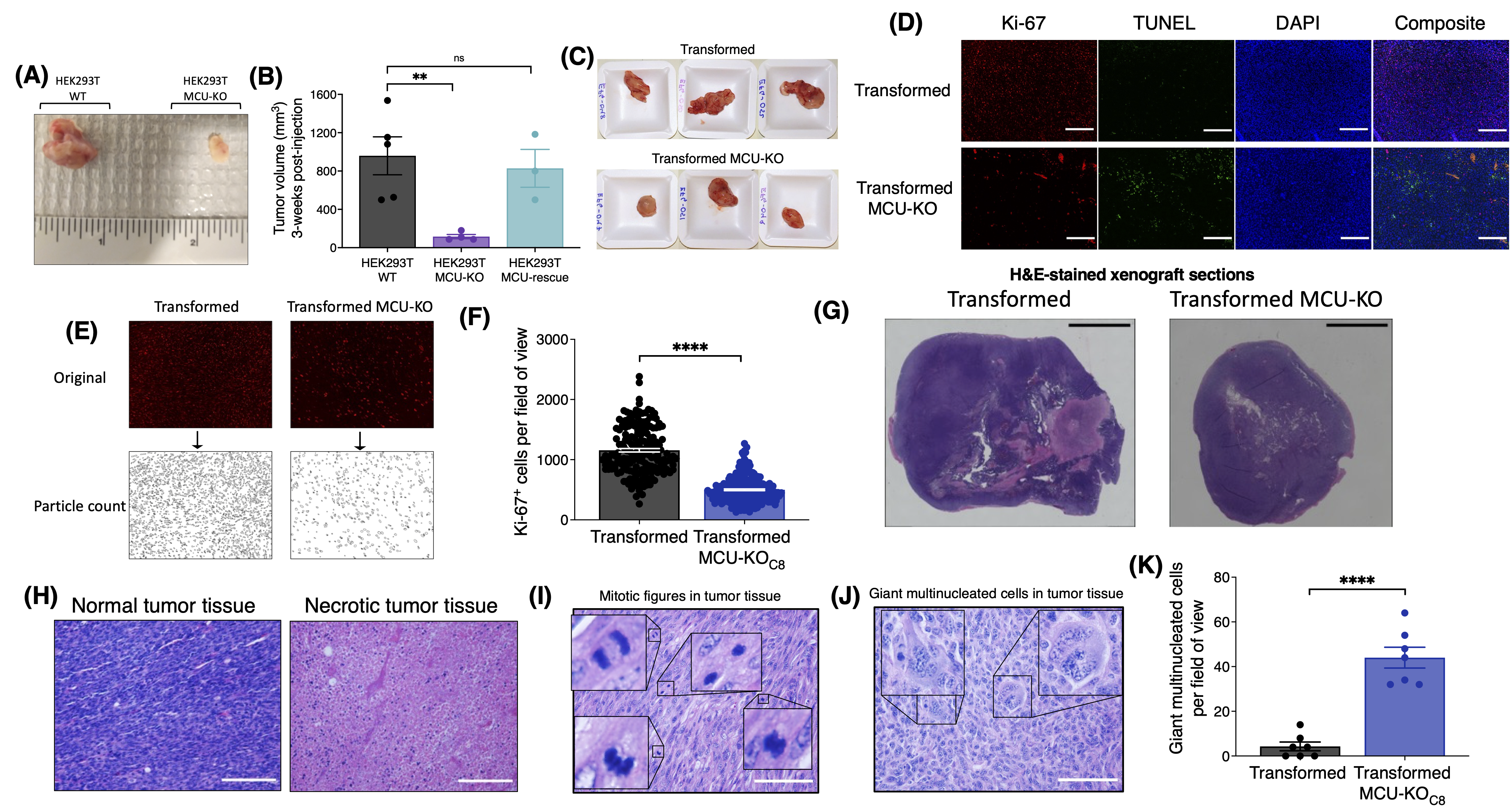


**Supplementary Figure 2.** Image processing and analyses of tumor xenografts. (**A**) Representative tumor xenografts derived from HEK293T WT and MCU-KO cells. (**B**) Volumes of HEK293T WT (n = 5), MCU-KO (n = 4), and MCU-rescue (n = 3) tumor xenografts 3 weeks post-injection (mean ± SEM, **p < 0.005, ns = non-significant, one-way ANOVA). (**C**) Representative images of whole tumor xenografts of transformed and MCU-KO fibroblasts. (**D**) Representative immunofluorescence (IF) images of tumor xenograft sections stained for Ki-67, TUNEL, and DAPI (scale bar, 200 μm). (**E**) Representative image of ki-67-stained tumor xenograft at magnification of 20x. (**F**) Quantification of ki-67^+^ cells in tumor xenografts of transformed and MCU-KO fibroblasts (mean ± SEM, n = 160, ****p < 0.0001, Student’s t-test). (**G**) Representative images of whole tumor xenografts of transformed and MCU-KO fibroblasts stained with H&E. The whole tumor was imaged at 4x, then all images were stitched together to form a single tumor image (scale bar, 1”; magnification 4x). (**H**) Representative images of hematoxylin and eosin (H&E) stained sections from tumor xenografts emphasizing normal (left) and necrotic (right) tissue (scale bar, 200 μm). (**I**) Representative H&E-stained xenograft section (scale bar, 200 μm) showing mitotic figures delineated by black squares. (**J**) Representative H&E staining of xenograft section emphasizing giant multinucleated cells outlined by black squares (scale bar, 200 μm). (**K**) Quantification of giant multinucleated cells in tumor xenografts. For each tumor, 10 different fields of view were examined as described in (G) (mean ± SEM, n = 7, ****p < 0.0001, Student’s t-test).

##
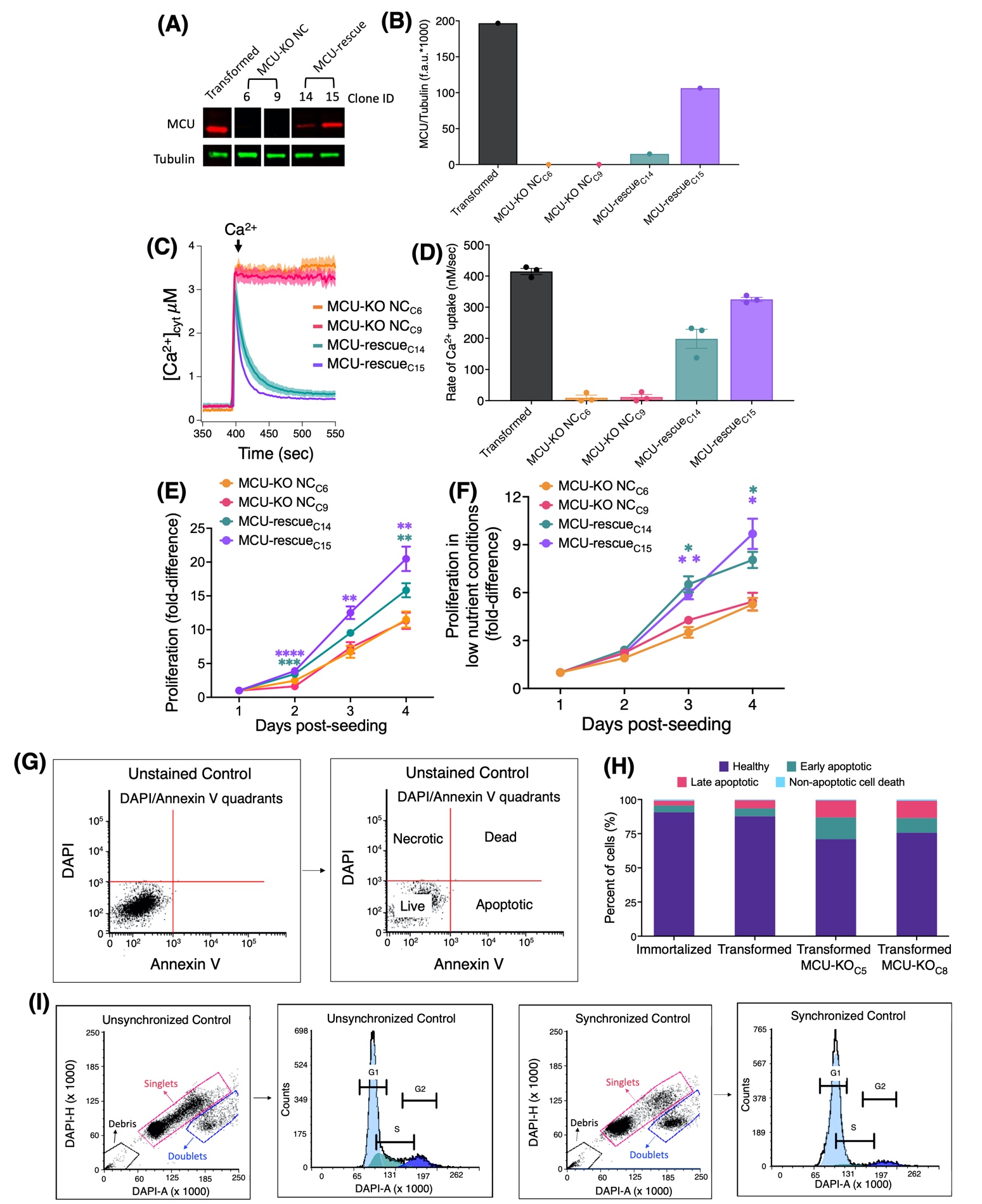


## Supplementary Figure 3. Rescue of MCU expression increases proliferation of transformed fibroblasts. (A) Representative immunoblots of MCU and tubulin in clones of MCU-KO negative controls (NC) and MCU-rescue transformed fibroblasts. (B) MCU levels normalized to tubulin. Values expressed as fluorescence units (a.u) (n = 1). (C) Averaged traces of [Ca^2+^]_cyt_ in suspensions of permeabilized MCU-KO NC and MCU-rescue transformed fibroblasts. (D) Mitochondrial Ca^2+^ uptake rates of permeabilized MCU-KO NC and MCU-rescue transformed fibroblasts (mean ± SEM, n = 3, ****p < 0.0001, ns = non-significant, one-way ANOVA). (E) Cell proliferation of MCU-KO NC and MCU-rescue clones. Each data point represents 3 biological replicates in triplicate. Fold-difference represents number of cells normalized to day 1 post-seeding. (mean ± SEM, n = 3, **p < 0.01, ***p < 0.001, ****p < 0.0001, two-way ANOVA compared with MCU-KO NC clone 6). (F) Cell proliferation of MCU-KO NC and MCU-rescue transformed fibroblasts in low-nutrient conditions. Each data point represents at least 3 biological replicates in triplicate, MCU-KO NC Clone 6 (n = 4), MCU-KO NC Clone 9 (n = 3), MCU-rescue Clone 14 (n = 3), MCU-rescue Clone 15 (n = 4). Fold-difference represents number of cells normalized to day 1 post-seeding. (mean ± SEM, *p < 0.05, **p < 0.01, two-way ANOVA compared with MCU-KO NC clone 6). (G) Gating strategy used for the analysis of annexin-V/DAPI flow cytometry data. First, live cells were gated in a forward and side-scatter plot. Then, singlets were gated in a forward-area and forward-height scatter plot. Finally, a 1 μM staurosporine-treated control was used to establish live, dead, and apoptotic quadrants in a DAPI and Annexin V scatter plot. (H) Percent (%) healthy cells, early apoptotic cells, late apoptotic/dead cells, and necrotic cells of annexin-V/DAPI FACS plot (mean, n = 3). (I) Gating strategy for the analysis of cell cycle flow cytometry data, same gating strategy as in (G). Unsynchronized and 15 μM lovastatin-synchronized samples were used as controls to validate the G1, S, and G2 peaks identified using Multicycle software for cell cycle analysis.

##
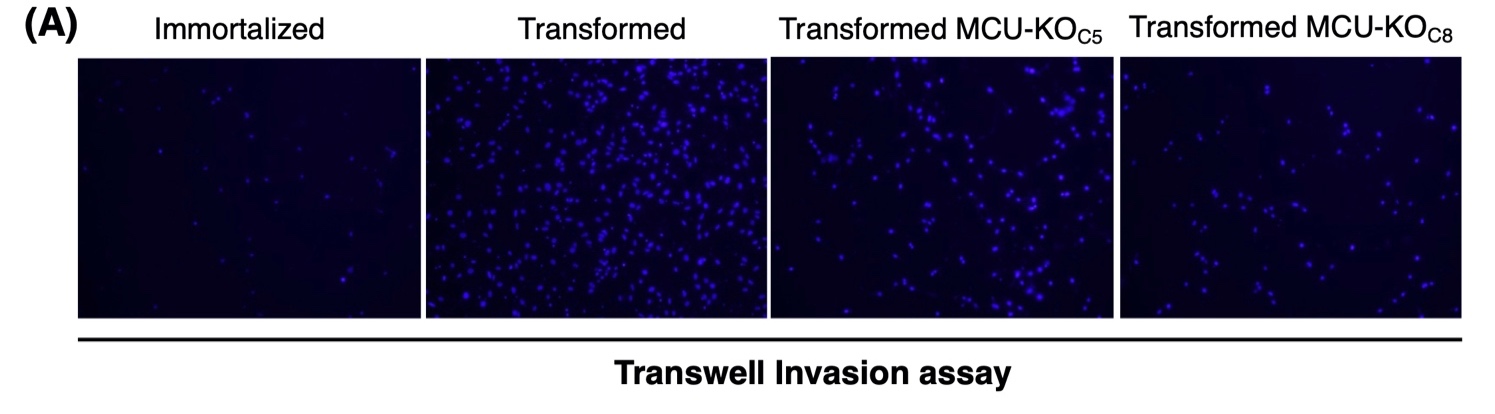


**Supplementary Figure 4.** Matrigel invasion of immortalized and transformed fibroblasts. **(A)** Representative fluorescent images of Hoechst 33342-stained nuclei in Transwell inserts during invasion assay.

**
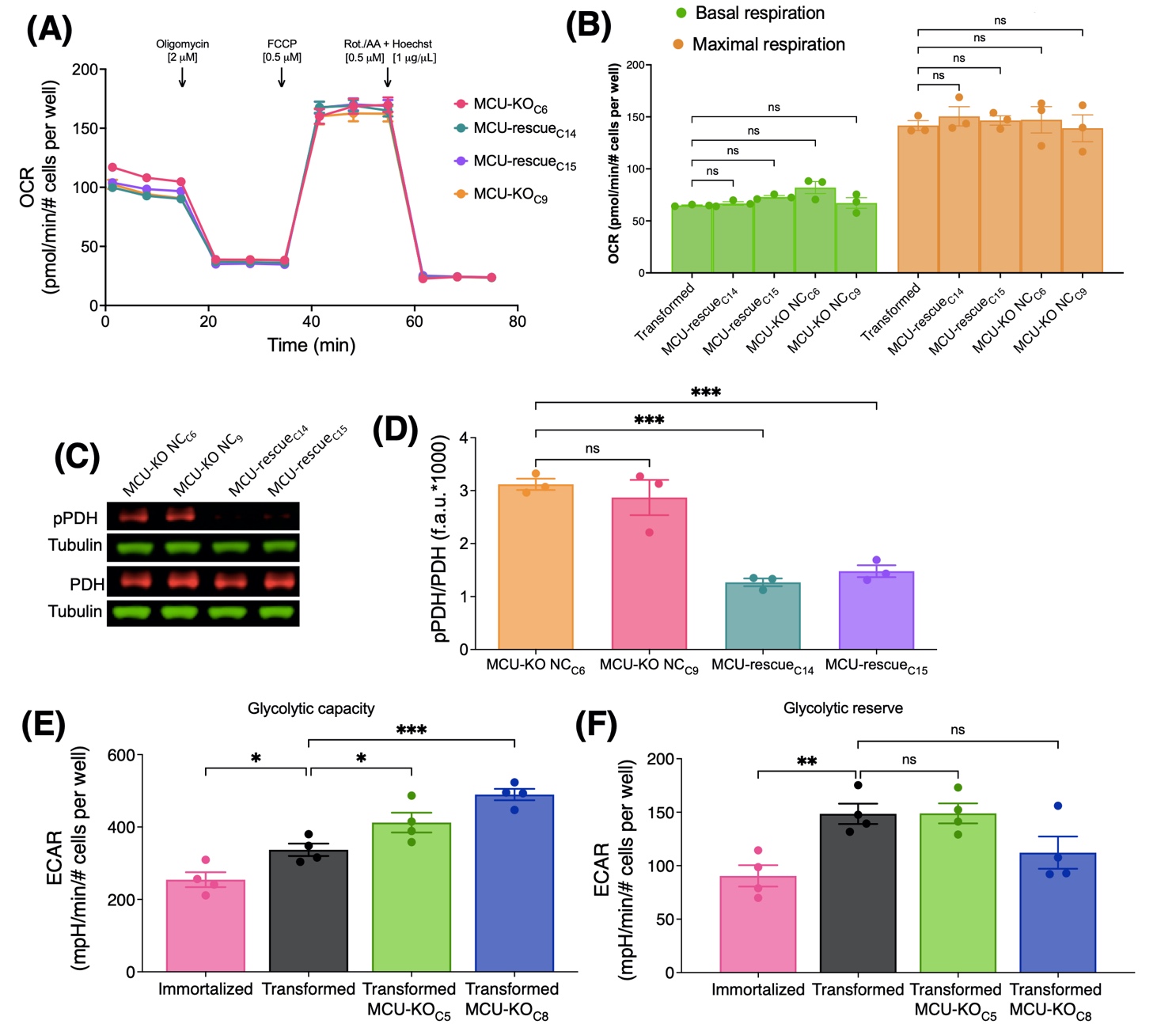
**

**Supplementary Figure 5.** Effects of MCU deletion on metabolic parameters in transformed fibroblasts. (**A**) Oxygen consumption rates (OCR) of MCU-KO NC and MCU-rescue transformed fibroblasts. Mean values compared to those of transformed fibroblast MCU-KO NC Clone 6 (mean ± SEM, n = 3, two-way). (**B**) Basal (green bars) and maximal (orange bars) respiration of MCU-KO NC and MCU-rescue transformed fibroblasts (mean ± SEM, n = 3, ns = non-significant, two-way ANOVA). (**C**) Representative immunoblots of PDH, pPDH and tubulin in MCU-KO NC and MCU-rescue transformed fibroblasts. (**D**) Relative protein levels of pPDH and PDH determined by measuring intensities of bands normalized to corresponding tubulin band intensity on same blot (mean ± SEM, n = 3, ***p < 0.001, ns = non-significant, one-way ANOVA). (**E**) Glycolytic capacity of immortalized and transformed fibroblasts (mean ± SEM, n = 4, *p < 0.05, ***p < 0.001, one-way ANOVA). (**F**) Glycolytic reserve of immortalized and transformed fibroblasts (mean ± SEM, n = 4, **p < 0.01, ns = non-significant, one-way ANOVA).

**Table 1.** Metabolites included in ^13^C_6_ D-Glucose metabolic tracing analysis.

| **Date** | **Compound** | **C_Label** | **Replicate ID** | **WT** | **MCU-KO NC C9** | **MCU-rescue C15** |
| --- | --- | --- | --- | --- | --- | --- |
| 10072022 | aspartate | **2** | 1 | 0.064 | 0.090 | 0.076 |
| 10072022 | aspartate | **2** | 2 | 0.083 | 0.071 | 0.108 |
| 10072022 | aspartate | **2** | 3 | 0.060 | 0.038 | 0.077 |
| 10122022 | aspartate | **2** | 4 | 0.058 | 0.061 | 0.114 |
| 10122022 | aspartate | **2** | 5 | 0.069 | 0.076 | 0.113 |
| 10122022 | aspartate | **2** | 6 | 0.062 | 0.037 | 0.083 |
| 10132022 | aspartate | **2** | 7 | 0.076 | 0.068 | 0.100 |
| 10132022 | aspartate | **2** | 8 | 0.067 | 0.068 | 0.106 |
| 10132022 | aspartate | **2** | 9 | 0.061 | 0.041 | 0.082 |
| 10072022 | aspartate | **3** | 1 | 0.034 | 0.047 | 0.104 |
| 10072022 | aspartate | **3** | 2 | 0.032 | 0.057 | 0.111 |
| 10072022 | aspartate | **3** | 3 | 0.037 | 0.045 | 0.115 |
| 10122022 | aspartate | **3** | 4 | 0.033 | 0.065 | 0.109 |
| 10122022 | aspartate | **3** | 5 | 0.028 | 0.054 | 0.115 |
| 10122022 | aspartate | **3** | 6 | 0.038 | 0.041 | 0.130 |
| 10132022 | aspartate | **3** | 7 | 0.034 | 0.045 | 0.092 |
| 10132022 | aspartate | **3** | 8 | 0.029 | 0.063 | 0.105 |
| 10132022 | aspartate | **3** | 9 | 0.036 | 0.043 | 0.126 |
| 10072022 | citrate-isocitrate | **2** | 1 | 0.260 | 0.354 | 0.288 |
| 10072022 | citrate-isocitrate | **2** | 2 | 0.292 | 0.356 | 0.344 |
| 10072022 | citrate-isocitrate | **2** | 3 | 0.260 | 0.283 | 0.385 |
| 10122022 | citrate-isocitrate | **2** | 4 | 0.308 | 0.391 | 0.294 |
| 10122022 | citrate-isocitrate | **2** | 5 | 0.320 | 0.390 | 0.353 |
| 10122022 | citrate-isocitrate | **2** | 6 | 0.299 | 0.270 | 0.340 |
| 10132022 | citrate-isocitrate | **2** | 7 | 0.305 | 0.399 | 0.309 |
| 10132022 | citrate-isocitrate | **2** | 8 | 0.306 | 0.381 | 0.373 |
| 10132022 | citrate-isocitrate | **2** | 9 | 0.345 | 0.274 | 0.405 |
| 10072022 | fumarate | **2** | 1 | 0.038 | 0.052 | 0.079 |
| 10072022 | fumarate | **2** | 2 | 0.066 | 0.054 | 0.096 |
| 10072022 | fumarate | **2** | 3 | 0.061 | 0.049 | 0.069 |
| 10122022 | fumarate | **2** | 4 | 0.046 | 0.064 | 0.084 |
| 10122022 | fumarate | **2** | 5 | 0.031 | 0.053 | 0.076 |
| 10122022 | fumarate | **2** | 6 | 0.076 | 0.038 | 0.057 |
| 10132022 | fumarate | **2** | 7 | 0.074 | 0.060 | 0.081 |
| 10132022 | fumarate | **2** | 8 | 0.070 | 0.055 | 0.091 |
| 10132022 | fumarate | **2** | 9 | 0.065 | 0.045 | 0.067 |
| 10072022 | fumarate | **3** | 1 | 0* | 0.031 | 0.084 |
| 10072022 | fumarate | **3** | 2 | 0.006 | 0.025 | 0.067 |
| 10072022 | fumarate | **3** | 3 | 0.044 | 0.032 | 0.078 |
| 10122022 | fumarate | **3** | 4 | 0.011 | 0.062 | 0.083 |
| 10122022 | fumarate | **3** | 5 | 0* | 0.040 | 0.081 |
| 10122022 | fumarate | **3** | 6 | 0.010 | 0.037 | 0.117 |
| 10132022 | fumarate | **3** | 7 | 0.010 | 0.053 | 0.096 |
| 10132022 | fumarate | **3** | 8 | 0.026 | 0.039 | 0.092 |
| 10132022 | fumarate | **3** | 9 | 0.033 | 0.034 | 0.074 |
| 10072022 | glucose | **6** | 1 | 0.865 | 0.929 | 0.928 |
| 10072022 | glucose | **6** | 2 | 0.982 | 0.982 | 0.949 |
| 10072022 | glucose | **6** | 3 | 0.715 | 0.657 | 0.802 |
| 10122022 | glucose | **6** | 4 | 0.755 | 0.945 | 0.928 |
| 10122022 | glucose | **6** | 5 | 0.981 | 0.995 | 0.937 |
| 10122022 | glucose | **6** | 6 | 0.830 | 0.663 | 0.727 |
| 10132022 | glucose | **6** | 7 | 0.808 | 0.870 | 0.894 |
| 10132022 | glucose | **6** | 8 | 0.977 | 0.990 | 0.962 |
| 10132022 | glucose | **6** | 9 | 0.634 | 0.624 | 0.698 |
| 10072022 | glutamate | **2** | 1 | 0.110 | 0.095 | 0.120 |
| 10072022 | glutamate | **2** | 2 | 0.117 | 0.086 | 0.148 |
| 10072022 | glutamate | **2** | 3 | 0.093 | 0.063 | 0.117 |
| 10122022 | glutamate | **2** | 4 | 0.107 | 0.094 | 0.125 |
| 10122022 | glutamate | **2** | 5 | 0.112 | 0.085 | 0.150 |
| 10122022 | glutamate | **2** | 6 | 0.109 | 0.066 | 0.122 |
| 10132022 | glutamate | **2** | 7 | 0.111 | 0.109 | 0.123 |
| 10132022 | glutamate | **2** | 8 | 0.116 | 0.102 | 0.151 |
| 10132022 | glutamate | **2** | 9 | 0.108 | 0.064 | 0.117 |
| 10072022 | glycine | **2** | 1 | 0.054 | 0.154 | 0.063 |
| 10072022 | glycine | **2** | 2 | 0.047 | 0.142 | 0.081 |
| 10072022 | glycine | **2** | 3 | 0.047 | 0.153 | 0.088 |
| 10122022 | glycine | **2** | 4 | 0.061 | 0.161 | 0.063 |
| 10122022 | glycine | **2** | 5 | 0.053 | 0.146 | 0.083 |
| 10122022 | glycine | **2** | 6 | 0.051 | 0.141 | 0.100 |
| 10132022 | glycine | **2** | 7 | 0.061 | 0.168 | 0.073 |
| 10132022 | glycine | **2** | 8 | 0.061 | 0.168 | 0.090 |
| 10132022 | glycine | **2** | 9 | 0.049 | 0.150 | 0.084 |
| 10072022 | lactate | **3** | 1 | 0.235 | 0.416 | 0.280 |
| 10072022 | lactate | **3** | 2 | 0.218 | 0.322 | 0.421 |
| 10072022 | lactate | **3** | 3 | 0.313 | 0.551 | 0.425 |
| 10122022 | lactate | **3** | 4 | 0.284 | 0.257 | 0.248 |
| 10122022 | lactate | **3** | 5 | 0.145 | 0.323 | 0.436 |
| 10122022 | lactate | **3** | 6 | 0.355 | 0.550 | 0.509 |
| 10132022 | lactate | **3** | 7 | 0.340 | 0.557 | 0.209 |
| 10132022 | lactate | **3** | 8 | 0.232 | 0.385 | 0.431 |
| 10132022 | lactate | **3** | 9 | 0.300 | 0.549 | 0.539 |
| 10072022 | malate | **2** | 1 | 0.061 | 0* | 0.037 |
| 10072022 | malate | **2** | 2 | 0.071 | 0.065 | 0.093 |
| 10072022 | malate | **2** | 3 | 0.049 | 0.036 | 0.072 |
| 10122022 | malate | **2** | 4 | 0.063 | 0.028 | 0* |
| 10122022 | malate | **2** | 5 | 0.063 | 0* | 0.096 |
| 10122022 | malate | **2** | 6 | 0.057 | 0.037 | 0.078 |
| 10132022 | malate | **2** | 7 | 0.059 | 0.069 | 0* |
| 10132022 | malate | **2** | 8 | 0.066 | 0.072 | 0.090 |
| 10132022 | malate | **2** | 9 | 0.058 | 0.039 | 0.077 |
| 10072022 | malate | **3** | 1 | 0.027 | 0* | 0* |
| 10072022 | malate | **3** | 2 | 0.032 | 0.045 | 0.093 |
| 10072022 | malate | **3** | 3 | 0.036 | 0.045 | 0.120 |
| 10122022 | malate | **3** | 4 | 0.030 | 0* | 0* |
| 10122022 | malate | **3** | 5 | 0.032 | 0.049 | 0.097 |
| 10122022 | malate | **3** | 6 | 0.034 | 0.045 | 0.118 |
| 10132022 | malate | **3** | 7 | 0.031 | 0.043 | 0.099 |
| 10132022 | malate | **3** | 8 | 0.031 | 0.044 | 0.092 |
| 10132022 | malate | **3** | 9 | 0.035 | 0.043 | 0.118 |
| 10072022 | pyruvate | **3** | 1 | 0.240 | 0.520 | 0.349 |
| 10072022 | pyruvate | **3** | 2 | 0.297 | 0.563 | 0.514 |
| 10072022 | pyruvate | **3** | 3 | 0.316 | 0.584 | 0.564 |
| 10122022 | pyruvate | **3** | 4 | 0.278 | 0.581 | 0.352 |
| 10122022 | pyruvate | **3** | 5 | 0.290 | 0.561 | 0.545 |
| 10122022 | pyruvate | **3** | 6 | 0.336 | 0.590 | 0.600 |
| 10132022 | pyruvate | **3** | 7 | 0.299 | 0.597 | 0.377 |
| 10132022 | pyruvate | **3** | 8 | 0.333 | 0.581 | 0.512 |
| 10132022 | pyruvate | **3** | 9 | 0.323 | 0.581 | 0.613 |
| 10072022 | serine | **3** | 1 | 0.050 | 0.177 | 0.080 |
| 10072022 | serine | **3** | 2 | 0.059 | 0.178 | 0.127 |
| 10072022 | serine | **3** | 3 | 0.045 | 0.179 | 0.113 |
| 10122022 | serine | **3** | 4 | 0.058 | 0.215 | 0.080 |
| 10122022 | serine | **3** | 5 | 0.057 | 0.184 | 0.141 |
| 10122022 | serine | **3** | 6 | 0.057 | 0.185 | 0.141 |
| 10132022 | serine | **3** | 7 | 0.060 | 0.217 | 0.094 |
| 10132022 | serine | **3** | 8 | 0.072 | 0.214 | 0.147 |
| 10132022 | serine | **3** | 9 | 0.057 | 0.187 | 0.135 |
|  |  |  |  | **0* -> no signal (area under curve = 0)** | | |

**Table 2.** Metabolites included in ^13^C_5_ L-glutamine metabolic tracing analysis.

| **Date** | **Compound** | **C_Label** | **Replicate ID** | **WT** | **MCU-KO NC C9** | **MCU-rescue C15** |
| --- | --- | --- | --- | --- | --- | --- |
| 10072022 | 4-aminobutyrate | **4** | 1 | 0.736 | 0.774 | 0.704 |
| 10072022 | 4-aminobutyrate | **4** | 2 | 0.766 | 0.773 | 0.746 |
| 10072022 | 4-aminobutyrate | **4** | 3 | 0.772 | 0.788 | 0.754 |
| 10122022 | 4-aminobutyrate | **4** | 4 | 0.758 | 0.770 | 0.739 |
| 10122022 | 4-aminobutyrate | **4** | 5 | 0.763 | 0.801 | 0.742 |
| 10122022 | 4-aminobutyrate | **4** | 6 | 0.761 | 0.792 | 0.751 |
| 10132022 | 4-aminobutyrate | **4** | 7 | 0.788 | 0.762 | 0.731 |
| 10132022 | 4-aminobutyrate | **4** | 8 | 0.744 | 0.782 | 0.742 |
| 10132022 | 4-aminobutyrate | **4** | 9 | 0.773 | 0.785 | 0.766 |
| 10072022 | aspartate | **4** | 1 | 0.529 | 0.598 | 0.430 |
| 10072022 | aspartate | **4** | 2 | 0.554 | 0.628 | 0.469 |
| 10072022 | aspartate | **4** | 3 | 0.595 | 0.618 | 0.524 |
| 10122022 | aspartate | **4** | 4 | 0.506 | 0.570 | 0.442 |
| 10122022 | aspartate | **4** | 5 | 0.550 | 0.604 | 0.475 |
| 10122022 | aspartate | **4** | 6 | 0.594 | 0.621 | 0.507 |
| 10132022 | aspartate | **4** | 7 | 0.515 | 0.557 | 0.412 |
| 10132022 | aspartate | **4** | 8 | 0.540 | 0.579 | 0.475 |
| 10132022 | aspartate | **4** | 9 | 0.574 | 0.623 | 0.522 |
| 10072022 | citrate-isocitrate | **4** | 1 | 0.348 | 0.445 | 0.316 |
| 10072022 | citrate-isocitrate | **4** | 2 | 0.426 | 0.436 | 0.346 |
| 10072022 | citrate-isocitrate | **4** | 3 | 0.414 | 0.438 | 0.399 |
| 10122022 | citrate-isocitrate | **4** | 4 | 0.352 | 0.441 | 0.325 |
| 10122022 | citrate-isocitrate | **4** | 5 | 0.375 | 0.472 | 0.381 |
| 10122022 | citrate-isocitrate | **4** | 6 | 0.373 | 0.452 | 0.432 |
| 10132022 | citrate-isocitrate | **4** | 7 | 0.384 | 0.464 | 0.324 |
| 10132022 | citrate-isocitrate | **4** | 8 | 0.378 | 0.464 | 0.364 |
| 10132022 | citrate-isocitrate | **4** | 9 | 0.516 | 0.468 | 0.441 |
| 10072022 | citrate-isocitrate | **5** | 1 | 0.071 | 0.045 | 0.047 |
| 10072022 | citrate-isocitrate | **5** | 2 | 0.070 | 0.054 | 0.052 |
| 10072022 | citrate-isocitrate | **5** | 3 | 0.088 | 0.065 | 0.049 |
| 10122022 | citrate-isocitrate | **5** | 4 | 0.071 | 0.041 | 0.040 |
| 10122022 | citrate-isocitrate | **5** | 5 | 0.069 | 0.050 | 0.051 |
| 10122022 | citrate-isocitrate | **5** | 6 | 0.095 | 0.058 | 0.059 |
| 10132022 | citrate-isocitrate | **5** | 7 | 0.071 | 0.037 | 0.039 |
| 10132022 | citrate-isocitrate | **5** | 8 | 0.069 | 0.052 | 0.052 |
| 10132022 | citrate-isocitrate | **5** | 9 | 0.058 | 0.061 | 0.048 |
| 10072022 | fumarate | **4** | 1 | 0.652 | 0.708 | 0.540 |
| 10072022 | fumarate | **4** | 2 | 0.718 | 0.694 | 0.554 |
| 10072022 | fumarate | **4** | 3 | 0.704 | 0.673 | 0.630 |
| 10122022 | fumarate | **4** | 4 | 0.675 | 0.646 | 0.582 |
| 10122022 | fumarate | **4** | 5 | 0.764 | 0.735 | 0.580 |
| 10122022 | fumarate | **4** | 6 | 0.660 | 0.702 | 0.644 |
| 10132022 | fumarate | **4** | 7 | 0.726 | 0.714 | 0.515 |
| 10132022 | fumarate | **4** | 8 | 0.696 | 0.663 | 0.620 |
| 10132022 | fumarate | **4** | 9 | 0.718 | 0.722 | 0.634 |
| 10072022 | glutamate | **5** | 1 | 0.627 | 0.660 | 0.634 |
| 10072022 | glutamate | **5** | 2 | 0.639 | 0.728 | 0.635 |
| 10072022 | glutamate | **5** | 3 | 0.672 | 0.702 | 0.650 |
| 10122022 | glutamate | **5** | 4 | 0.622 | 0.649 | 0.581 |
| 10122022 | glutamate | **5** | 5 | 0.638 | 0.717 | 0.638 |
| 10122022 | glutamate | **5** | 6 | 0.665 | 0.695 | 0.656 |
| 10132022 | glutamate | **5** | 7 | 0.636 | 0.649 | 0.595 |
| 10132022 | glutamate | **5** | 8 | 0.640 | 0.681 | 0.642 |
| 10132022 | glutamate | **5** | 9 | 0.669 | 0.702 | 0.650 |
| 10072022 | glutamine | **5** | 1 | 0.977 | 0.988 | 0.987 |
| 10072022 | glutamine | **5** | 2 | 0.986 | 0.986 | 0.975 |
| 10072022 | glutamine | **5** | 3 | 0.985 | 0.983 | 0.818 |
| 10122022 | glutamine | **5** | 4 | 0.977 | 0.986 | 0.988 |
| 10122022 | glutamine | **5** | 5 | 0.987 | 0.986 | 0.975 |
| 10122022 | glutamine | **5** | 6 | 0.985 | 0.986 | 0.986 |
| 10132022 | glutamine | **5** | 7 | 0.980 | 0.987 | 0.989 |
| 10132022 | glutamine | **5** | 8 | 0.989 | 0.986 | 0.984 |
| 10132022 | glutamine | **5** | 9 | 0.985 | 0.986 | 0.986 |
| 10072022 | malate | **4** | 1 | 0.493 | 0.635 | 0.479 |
| 10072022 | malate | **4** | 2 | 0.573 | 0.700 | 0.491 |
| 10072022 | malate | **4** | 3 | 0.607 | 0.596 | 0.560 |
| 10122022 | malate | **4** | 4 | 0.400 | 0.624 | 0.462 |
| 10122022 | malate | **4** | 5 | 0.554 | 0.613 | 0.504 |
| 10122022 | malate | **4** | 6 | 0.577 | 0.614 | 0.571 |
| 10132022 | malate | **4** | 7 | 0.550 | 0.593 | 0.436 |
| 10132022 | malate | **4** | 8 | 0.559 | 0.594 | 0.510 |
| 10132022 | malate | **4** | 9 | 0.601 | 0.611 | 0.568 |
